# Supplementary material for: The Dystrophin-Dystroglycan complex ensures cytokinesis efficiency in Drosophila epithelia
Source: EMBO Rep. 2024 Nov 15;26(2):307–28. doi: 10.1038/s44319-024-00319-y (PMC11772804; doi:10.1038/s44319-024-00319-y)
Supplement: Supplementary file 4 — Table EV1 [file 44319_2024_319_MOESM4_ESM.pdf]

Table EV1. Mutant alleles and transgenes

Mutant allele or transgene	Citation	Stock number or lab of origin
Dys^{Df}	(Christoforou *et al*, 2008)	BDSC #7663
Dys^{E17}	(Christoforou *et al.*, 2008)	BDSC #63047
Dg^{086}	(Christoforou *et al.*, 2008)	BDSC #63049
Dg^{043}	(Christoforou *et al.*, 2008)	BDSC #63048
$Dys^{MI025024}$	(Bellen *et al*, 2011)	BDSC #24182
$Dys^{long181}$	this study	Vincent Mirouse
Dys^{RE225}	this study	Vincent Mirouse
Dys^{short}:sfGFP	this study	Vincent Mirouse
Dys^{long}:sfGFP	this study	Vincent Mirouse
Dys:sfGFP	(Dennis *et al*, 2024)	Vincent Mirouse
UAS-Dys^{short}:GFP	this study	Vincent Mirouse
UAS-$Dys^{short\Delta SR24}$:GFP	this study	Vincent Mirouse
UAS-$Dys^{short\Delta SD}$:GFP	this study	Vincent Mirouse
UAS-$Dys^{short\Delta CT}$:GFP	this study	Vincent Mirouse
Dg:GFP	(Villedieu *et al.*, 2023)	Yohanns Bellaiche
UAS-Dg:GFP	(Bogdanik *et al*, 2008)	Marie-Laure Parmentier
GFP:aPKC	(Chen *et al*, 2018)	Daniel St Johnston
Zip:GFP	(Lowe *et al*, 2014)	DGGR #115082
ECad:GFP	(Pinheiro *et al.*, 2017)	Yohanns Bellaiche
Sqh:3xmKate2	(Pinheiro *et al.*, 2017)	Yohanns Bellaiche
Ed:GFP	(Lowe *et al.*, 2014)	DGGR # 115114
Collagen IV:GFP	(Morin *et al*, 2001)	BDSC #98343
LanB1:GFP	(Sarov *et al*, 2016)	VDRC #318180
LanA:GFP	(Sarov *et al.*, 2016)	VDRC #318155
Nrg:GFP	(Morin *et al.*, 2001)	BDSC #6844
Ubq-Tub:RFP	(Dobbelaere *et al*, 2008)	Jordan Raff
tub-Gal80^{ts}	(McGuire *et al.*, 2003)	BDSC #7018
tj-Gal4	(Olivieri *et al.*, 2010)	DGGR #104055
UAS-mRFP:Anillin	Donated to BDSC by Andy Wilde	BDSC #52220
UAS-ECad	(Pacquelet & Rorth, 2005)	BDSC #58494
UAS-Anillin RNAi$^{(KK107556)}$	www.vdrc.at	VDRC #104674
UAS-Tum RNAi$^{(TRIP.JF01639)}$	(Perkins *et al*, 2015)	BDSC # 28982
UAS-Myr:GFP	(Pfeiffer *et al*, 2010)	BDSC #32197
UAS-mCherry	http://www.flyrnai.org/TRiP-HOME.html	BDSC #35787
UAS-mCherry RNAi	http://www.flyrnai.org/TRiP-HOME.html	BDSC #35785
