# Supplementary material for: The Dystrophin-Dystroglycan complex ensures cytokinesis efficiency in Drosophila epithelia
Source: EMBO Rep. 2024 Nov 15;26(2):307–28. doi: 10.1038/s44319-024-00319-y (PMC11772804; doi:10.1038/s44319-024-00319-y)
Supplement: Supplementary file 5 — Table EV2 [file 44319_2024_319_MOESM5_ESM.pdf]

Table EV2. List of *Drosophila* genotypes

Figure	Genotype
**1B, 1C**	*tj-Gal4*, UAS-Anillin RNAi/+; UAS-Myr:GFP, tub-Gal80^{ts}/UAS-mCherry<br>*tj-Gal4*, UAS-Anillin RNAi/UAS-ECad; UAS-Myr:GFP, tub-Gal80^{ts}/+<br>*tj-Gal4*/UAS-ECad; UAS-Myr:GFP, tub-Gal80^{ts}/+
**1D, 1E, 1F, 1G**	*tj-Gal4*, UAS-Anillin RNAi/+; UAS-Myr:GFP, tub-Gal80^{ts}/UAS-mCherry<br>*tj-Gal4*, UAS-Anillin RNAi/UAS-CAM or UAS-ECM-protein RNAi; UAS-Myr:GFP, tub-Gal80^{ts}/+ or *tj-Gal4*, UAS-Anillin RNAi/+; UAS-Myr:GFP, tub-Gal80^{ts}/UAS-CAM or UAS-ECM-protein RNAi
**2B, 2C**	*tj-Gal4*, ECad:GFP/+; $Dys^{E17/Df}$<br>*tj-Gal4*, ECad:GFP/UAS-Anillin RNAi<br>*tj-Gal4*, ECad:GFP/UAS-Anillin RNAi; $Dys^{E17/Df}$<br>*tj-Gal4*, ECad:GFP/+; $Dys^{MI025024/Df}$<br>*tj-Gal4*, ECad:GFP/UAS-Anillin RNAi; $Dys^{MI025024/Df}$
**2D, 2E**	*tj-Gal4*/UAS-mRFP:Anillin<br>*tj-Gal4*/UAS-mRFP:Anillin; $Dys^{MI025024/Df}$<br>*tj-Gal4*/UAS-mRFP:Anillin; $Dys^{E17/Df}$
**3A**	*tj-Gal4*, UAS-Dg:GFP/UASp-mRFP:Anillin
**3B**	Dg:GFP/Sqh:3xmKate2
**3C, 3D**	Sqh:3xmKate2/+; Dys:sfGFP
**3E**	Tub:RFP/+; Dys:sfGFP
**4A**	GFP:aPKC/CyO<br>IF/CyO; Dys:sfGFP<br>Dys^{short}:sfGFP/TM3<br>Dys^{long}:sfGFP/TM3
**4B**	Sqh:3xmKate2/+; Dys^{short}:sfGFP<br>Sqh:3xmKate2/+; Dys^{long}:sfGFP
**4C, 4D**	*tj-Gal4*, ECad:GFP/+; MKRS/TM6<br>*tj-Gal4*, ECad:GFP/+; $Dys^{long181/Df}$<br>*tj-Gal4*, ECad:GFP/UAS-Anillin RNAi<br>*tj-Gal4*, ECad:GFP/UAS-Anillin RNAi; $Dys^{long181/Df}$<br>*tj-Gal4*, ECad:GFP/+; $Dys^{RE225/Df}$<br>*tj-Gal4*, ECad:GFP/UAS-Anillin RNAi; $Dys^{RE225/Df}$
**5A**	*tj-Gal4*, Zip:GFP/UAS-Anillin RNAi<br>*tj-Gal4*, Zip:GFP/UAS-Anillin RNAi; $Dys^{E17/Df}$

**5B**	*tj-Gal4*, Zip:GFP/+<br>*tj-Gal4*, Zip:GFP/UAS-Anillin RNAi<br>*tj-Gal4*, Zip:GFP/UAS-Anillin RNAi; $Dys^{E17/Df}$
**5C**	*tj-Gal4*, Zip:GFP/UAS-Anillin RNAi<br>*tj-Gal4*, Zip:GFP/UAS-Anillin RNAi; $Dys^{E17/Df}$<br>*tj-Gal4*, Zip:GFP/UAS-Anillin RNAi; $Dys^{long181/Df}$<br>*tj-Gal4*, Zip:GFP/UAS-Anillin RNAi; $Dys^{RE225/Df}$
**5D, 5E**	*tj-Gal4*, Sqh:3xmKate2/+<br>$Dg^{086/043}$; Sqh:3xmKate2/+<br>Sqh:3xmKate2/+; $Dys^{E17/Df}$
**5F**	*tj-Gal4*, Zip:GFP/+<br>*tj-Gal4*, Zip:GFP/+; $Dys^{long181/Df}$<br>*tj-Gal4*, Zip:GFP/+; $Dys^{R225/Df}$
**5G**	*tj-Gal4*, Sqh:3xmKate2/+<br>$Dg^{086/043}$; Sqh:3xmKate2/+<br>Sqh:3xmKate2/+; $Dys^{E17/Df}$<br>*tj-Gal4*, Zip:GFP/+<br>*tj-Gal4*, Zip:GFP/+; $Dys^{long181/Df}$<br>*tj-Gal4*, Zip:GFP/+; $Dys^{RE225/Df}$
**EV1A, EV1B**	*tj-Gal4*, UAS-Anillin RNAi/+; UAS-Myr:GFP, tub-$Gal80^{ts}$/UAS-mCherry
**EV2A**	*tj-Gal4*, ECad:GFP/+; UAS-mCherry RNAi/+<br>*tj-Gal4*, ECad:GFP/+; UAS-ECad RNAi/+
**EV2B**	*tj-Gal4*/+; UAS-mCherry/+<br>*tj-Gal4*/+; UAS-NCad RNAi/+
**EV2C**	*tj-Gal4*/+; tub-$Gal80^{ts}$/UAS-mCherry RNAi<br>*tj-Gal4*/+*;* tub-$Gal80^{ts}$/UAS-Fas2 RNAi
**EV2D**	*tj-Gal4*/+; tub-$Gal80^{ts}$/UAS-mCherry RNAi<br>*tj-Gal4*/+; tub-$Gal80^{ts}$/UAS-Fas3 RNAi
**EV2E**	*tj-Gal4*, Ed:GFP/+; UAS-mCherry RNAi<br>*tj-Gal4*, Ed:GFP/UAS-Ed RNAi; +/+
**EV2F**	Nrg:GFP/+; *tj-Gal4*/+<br>Nrg:GFP/+; *tj-Gal4*/+; UAS-Nrg RNAi/+
**EV2G**	*tj-Gal4*/+; UAS-mCherry/+<br>*tj-Gal4*/+; UAS-*βPS-integrin* RNAi/+

**EV2H**	*tj-Gal4*/Dg:GFP; UAS-mCherry RNAi/+<br>*tj-Gal4*/Dg:GFP; UAS-Dg RNAi/+
**EV2I**	*tj-Gal4*/+; LanA:GFP/UAS-mCherry<br>*tj-Gal4*/+; LanA:GFP/UAS-LanA RNAi
**EV2J**	*tj-Gal4*/+; LanB1:GFP/UAS-mCherry RNAi<br>*tj-Gal4*/+; LanB1:GFP/LanB1 RNAi
**EV2K**	*tj-Gal4*/+; UAS-mCherry/+<br>*tj-Gal4*/+; UAS-Perlecan RNAi/+
**EV2L**	*tj-Gal4*/Collagen IV:GFP; tub-Gal80^{ts}/+<br>*tj-Gal4*/Collagen IV:GFP; tub-Gal80^{ts}/UAS-Collagen IV RNAi
**EV3A**	*tj-Gal4*, ECad:GFP/+; UAS-mCherry RNAi/+<br>*tj-Gal4*, ECad:GFP/+; UAS-ECad RNAi/+
**EV3B**	*tj-Gal4*, UAS-Anillin RNAi/+; UAS-Myr:GFP tub-Gal80^{ts}/UAS-mCherry<br>*tj-Gal4*, UAS-Anillin RNAi/+; UAS-Myr:GFP tub-Gal80^{ts}/UAS-αPS1 RNAi #1<br>*tj-Gal4*, UAS-Anillin RNAi/+; UAS-Myr:GFP, tub-Gal80^{ts}/UAS-βPS RNAi #1<br>*tj-Gal4*, UAS-Anillin RNAi/+; UAS-Myr:GFP, tub-Gal80^{ts}/UAS-βPS RNAi #2<br>*tj-Gal4*, UAS-Anillin RNAi/+; UAS-Myr:GFP, tub-Gal80^{ts}/UAS-Dg RNAi<br>*tj-Gal4*, UAS-Anillin RNAi/UAS-Kug RNAi; UAS-Myr:GFP, tub-Gal80^{ts}/+<br>*tj-Gal4*, UAS-Anillin RNAi/+; UAS-Myr:GFP, tub-Gal80^{ts}/UAS-Collagen IV RNAi<br>*tj-Gal4*, UAS-Anillin RNAi/+; UAS-Myr:GFP, tub-Gal80^{ts}/UAS-Perlecan RNAi
**EV3C**	*tj-Gal4*/+; UAS-Myr:GFP, tub-Gal80^{ts}/UAS-mCherry<br>*tj-Gal4*/UAS-Kug RNAi; UAS-Myr:GFP, tub-Gal80^{ts}/+<br>*tj-Gal4*/UAS-Ed RNAi; UAS-Myr:GFP, tub-Gal80^{ts}/+<br>*tj-Gal4*/+; UAS-Myr:GFP, tub-Gal80^{ts}/UAS-Nrg RNAi #1<br>*tj-Gal4*/+; UAS-Myr:GFP, tub-Gal80^{ts}/UAS-NrxIV RNAi<br>*tj-Gal4*/+; UAS-Myr:GFP, tub-Gal80^{ts}/UAS-Dg RNAi<br>*tj-Gal4*/+; UAS-Myr:GFP, tub-Gal80^{ts}/UAS-Perlecan RNAi<br>*tj-Gal4*/+; UAS-Myr:GFP, tub-Gal80^{ts}/UAS-Cals RNAi
**EV3D**	*tj-Gal4*, ECad:GFP/Gal80^{ts}; UAS-mCherry RNAi/UAS-Dg RNAi<br>*tj-Gal4*, ECad:GFP/Gal80^{ts}; UAS-mCherry RNAi/UAS-Tum RNAi<br>*tj-Gal4*, ECad:GFP/Gal80^{ts}; UAS-Dg RNAi/UAS-Tum RNAi
**EV4C**	Sqh:3xmKate2/Dg:GFP
**EV4D**	Sqh:3xmKate2/+; Dys:GFP/+
**EV5A**	*Dys*^{short}:sfGFP/+
**EV5B**	*Dys*^{long}:sfGFP/+

**EV5C**	*tj-Gal4*, Sqh:3xmKate2/+; *Dys*^{short}:GFP/+<br>*tj-Gal4*, Sqh:3xmKate2/+; *Dys*$^{short\Delta SR24}$:GFP/+<br>*tj-Gal4*, Sqh:3xmKate2/+; *Dys*$^{short\Delta SD}$:GFP/+<br>*tj-Gal4*, Sqh:3xmKate2/+; *Dys*$^{short\Delta CT}$:GFP/+
**EV5D**	*tj-Gal4*, Sqh:3xmKate2/+; *Dys*^{short}:GFP/+
**EV5E**	*tj-Gal4*, Sqh:3xmKate2/+; *Dys*$^{short\Delta SR24}$:GFP/+
**EV5F**	*tj-Gal4*, Sqh:3xmKate2/+; *Dys*$^{short\Delta SD}$:GFP/+
**EV5G**	*tj-Gal4*, Sqh:3xmKate2/+; *Dys*$^{short\Delta CT}$:GFP/+
