# Supplementary figures and images for: The Dystrophin-Dystroglycan complex ensures cytokinesis efficiency in Drosophila epithelia
Source: EMBO Rep. 2024 Nov 15;26(2):307–28. doi: 10.1038/s44319-024-00319-y (PMC11772804; doi:10.1038/s44319-024-00319-y)

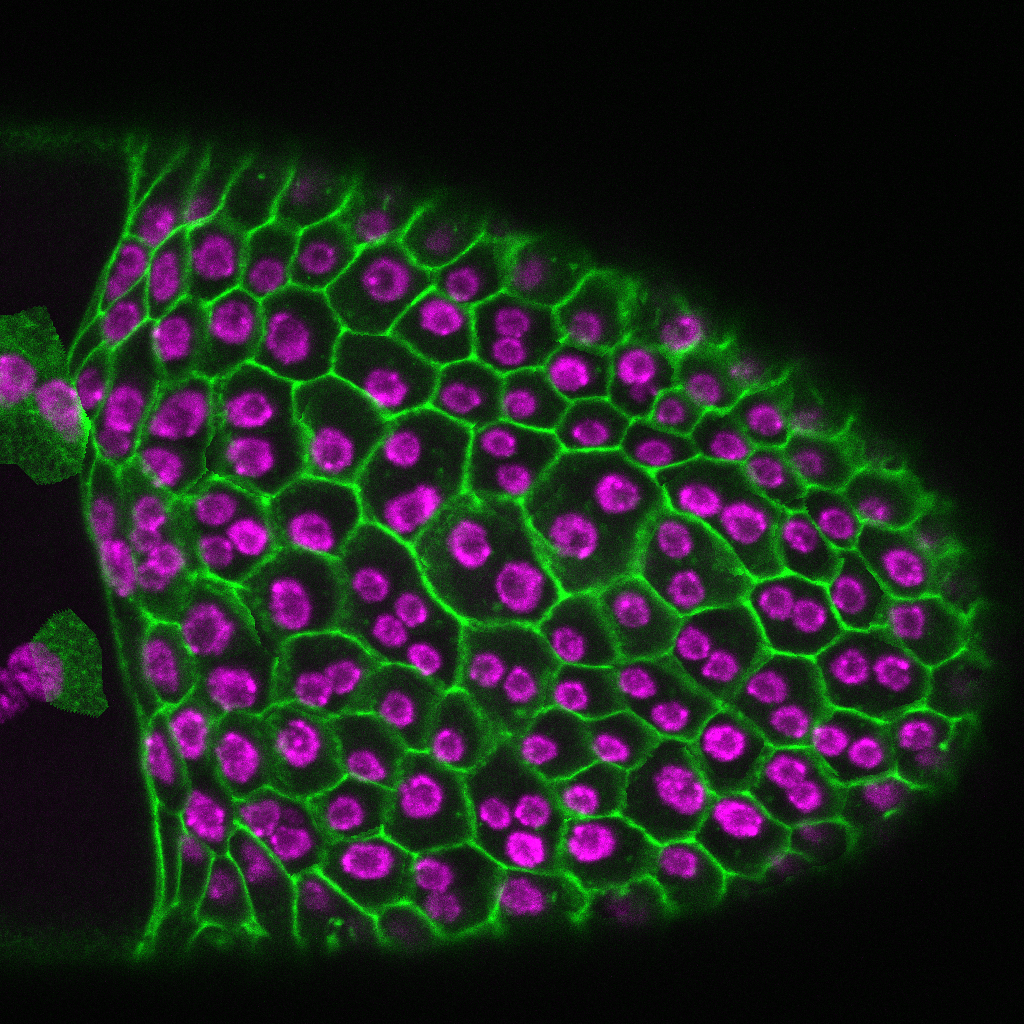

Supplement: Supplementary file 14 — Source data Fig. 1 [file 44319_2024_319_MOESM14_ESM.zip › Figure 1/1B/UASAniRNAi_UASECad_UASMyrGFP_DAPI.tif]

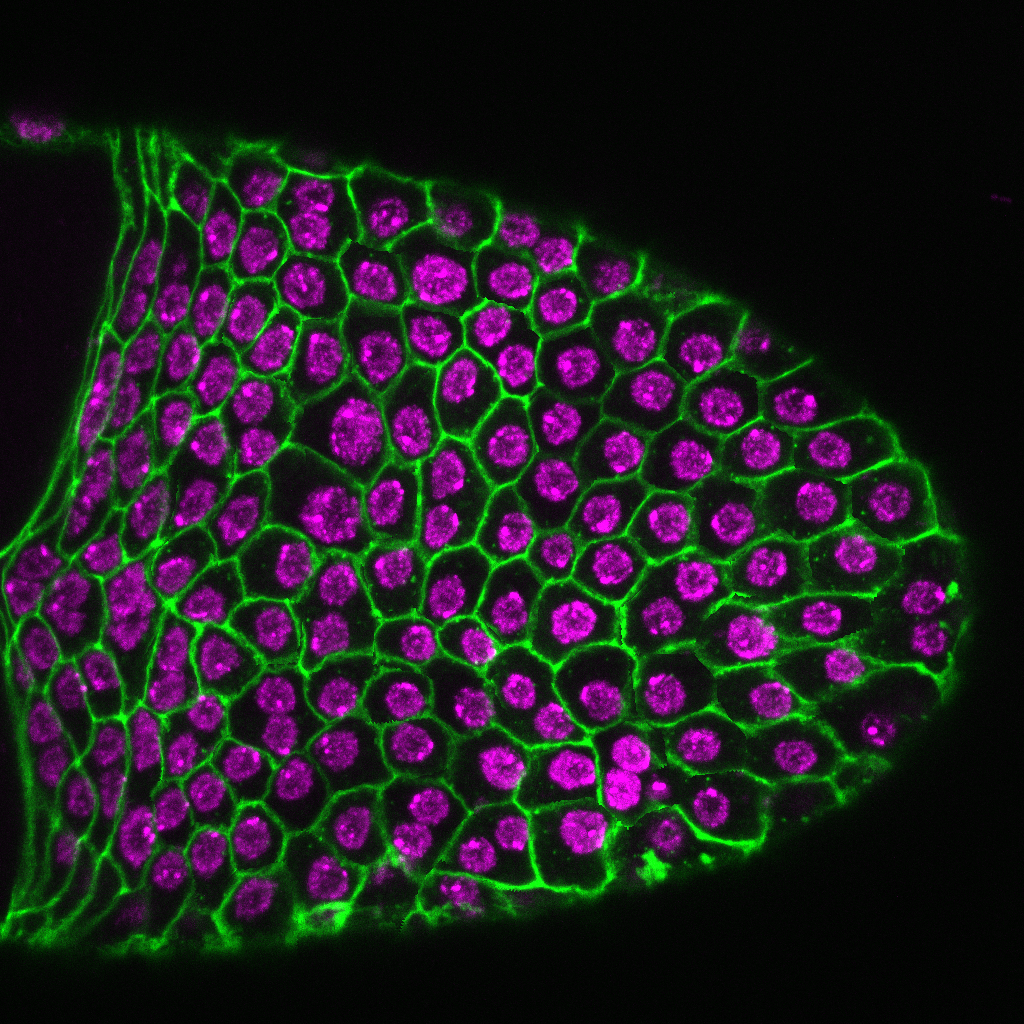

Supplement: Supplementary file 14 — Source data Fig. 1 [file 44319_2024_319_MOESM14_ESM.zip › Figure 1/1B/UASAniRNAi_UASmcherry_UASMyrGFP_DAPI.tif]

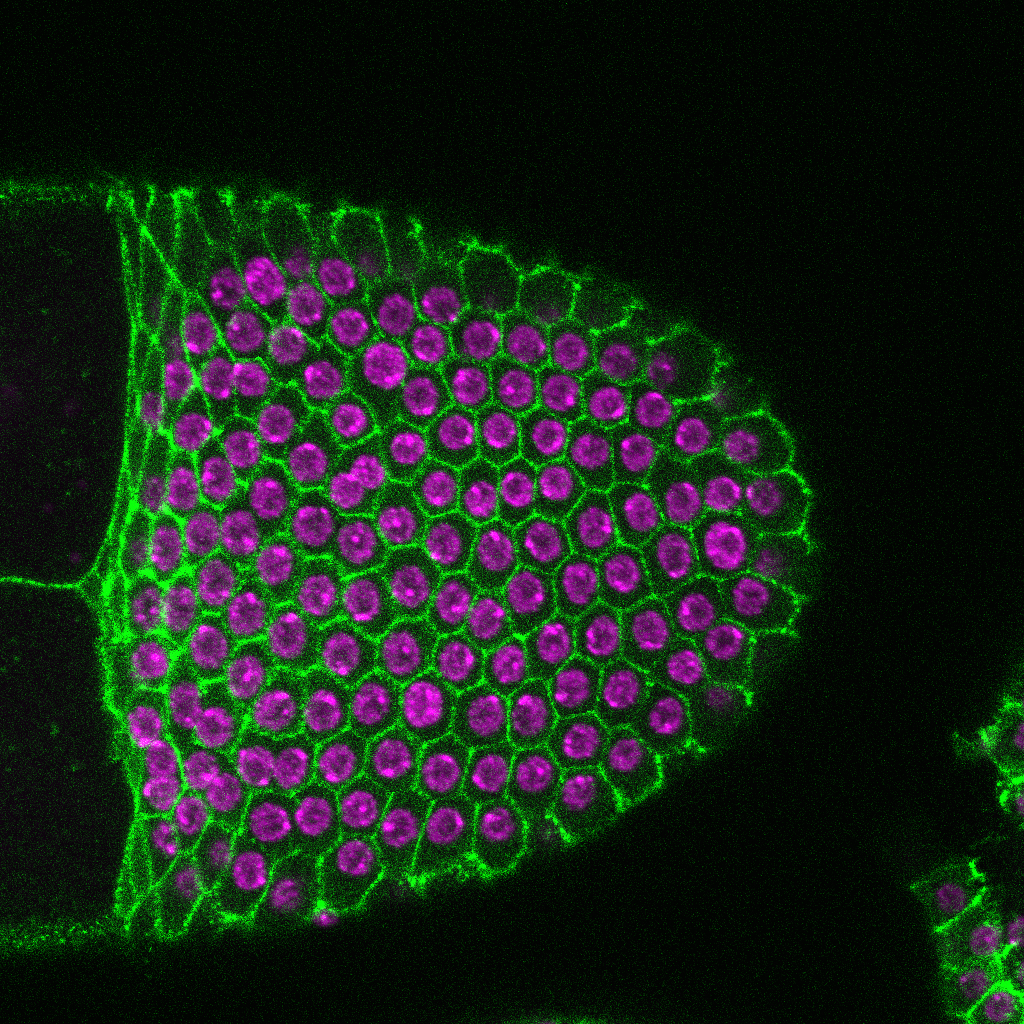

Supplement: Supplementary file 15 — Source data Fig. 2 [file 44319_2024_319_MOESM15_ESM.zip › Figure 2/2C/DysE17.tif]

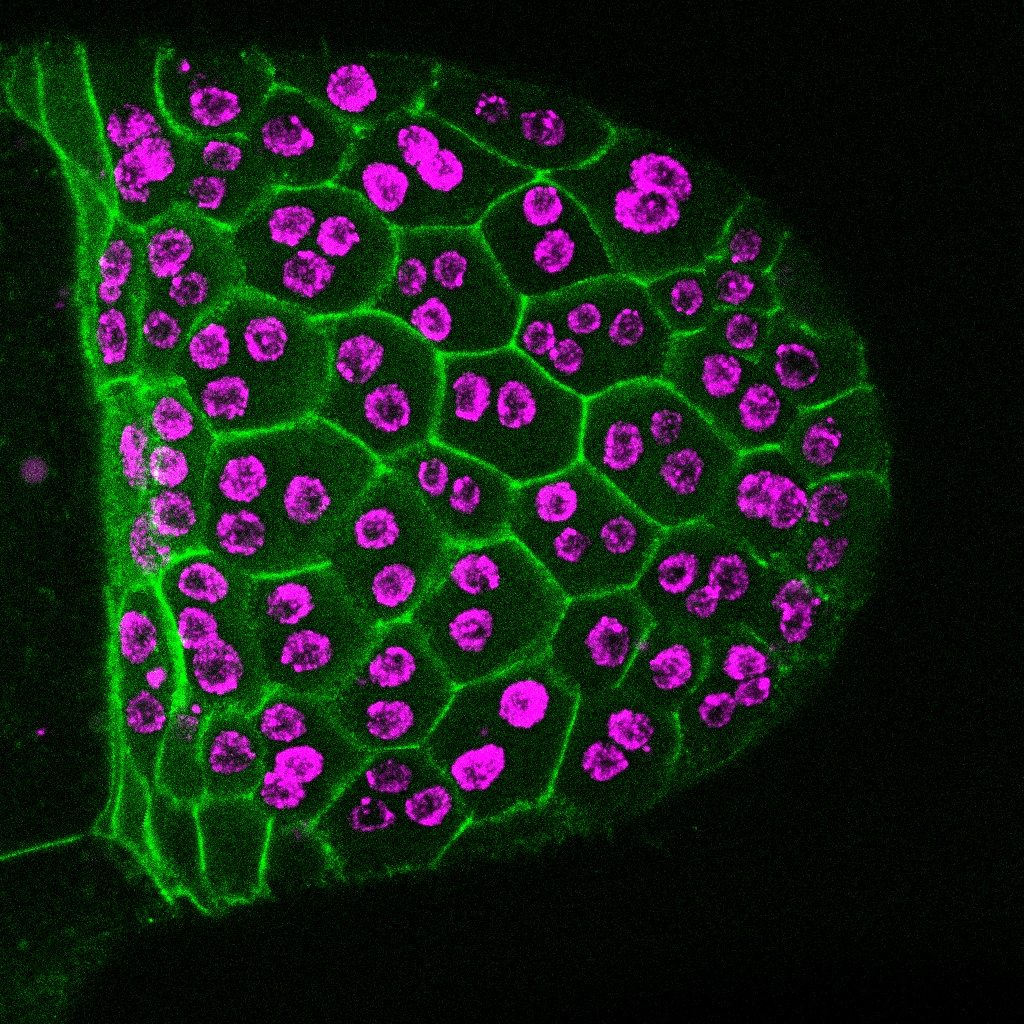

Supplement: Supplementary file 15 — Source data Fig. 2 [file 44319_2024_319_MOESM15_ESM.zip › Figure 2/2C/DysMI025024_AniRNAi.tif]

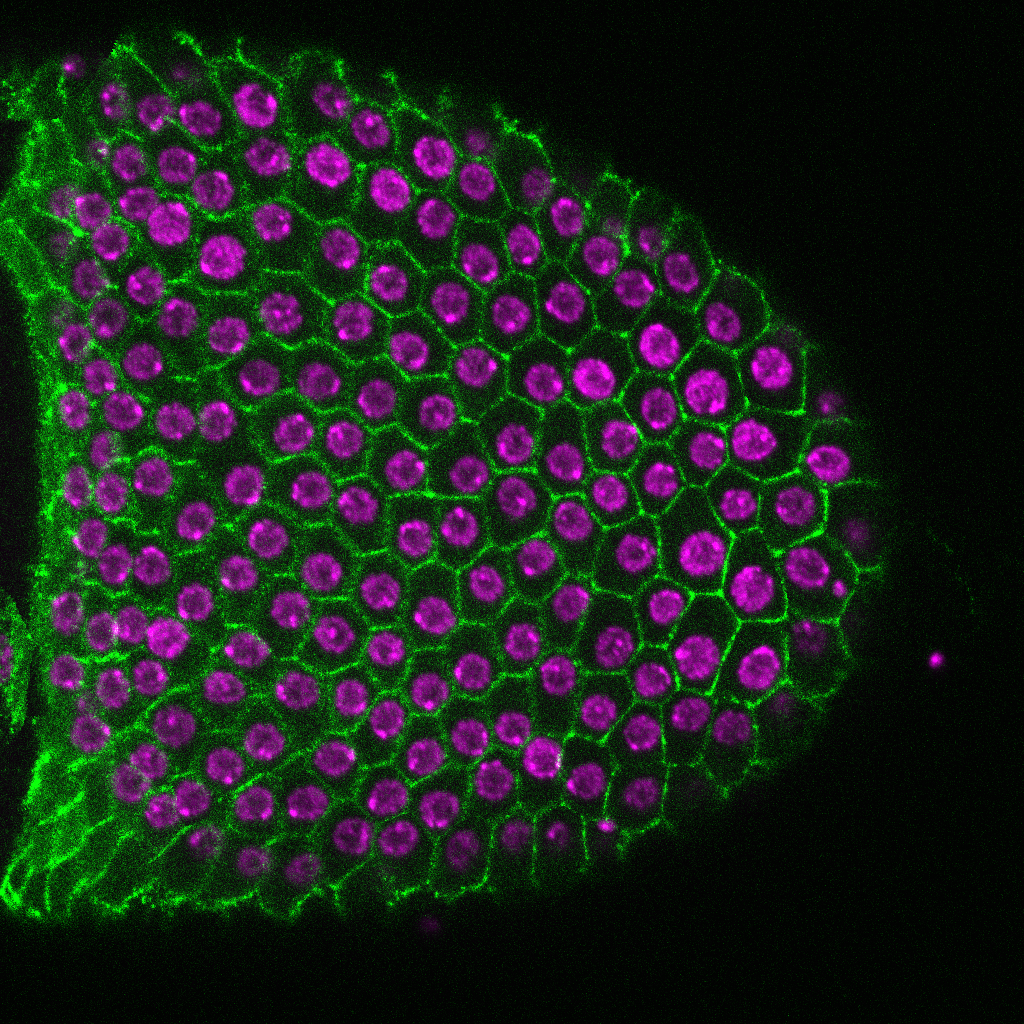

Supplement: Supplementary file 15 — Source data Fig. 2 [file 44319_2024_319_MOESM15_ESM.zip › Figure 2/2C/DysMI025024.tif]

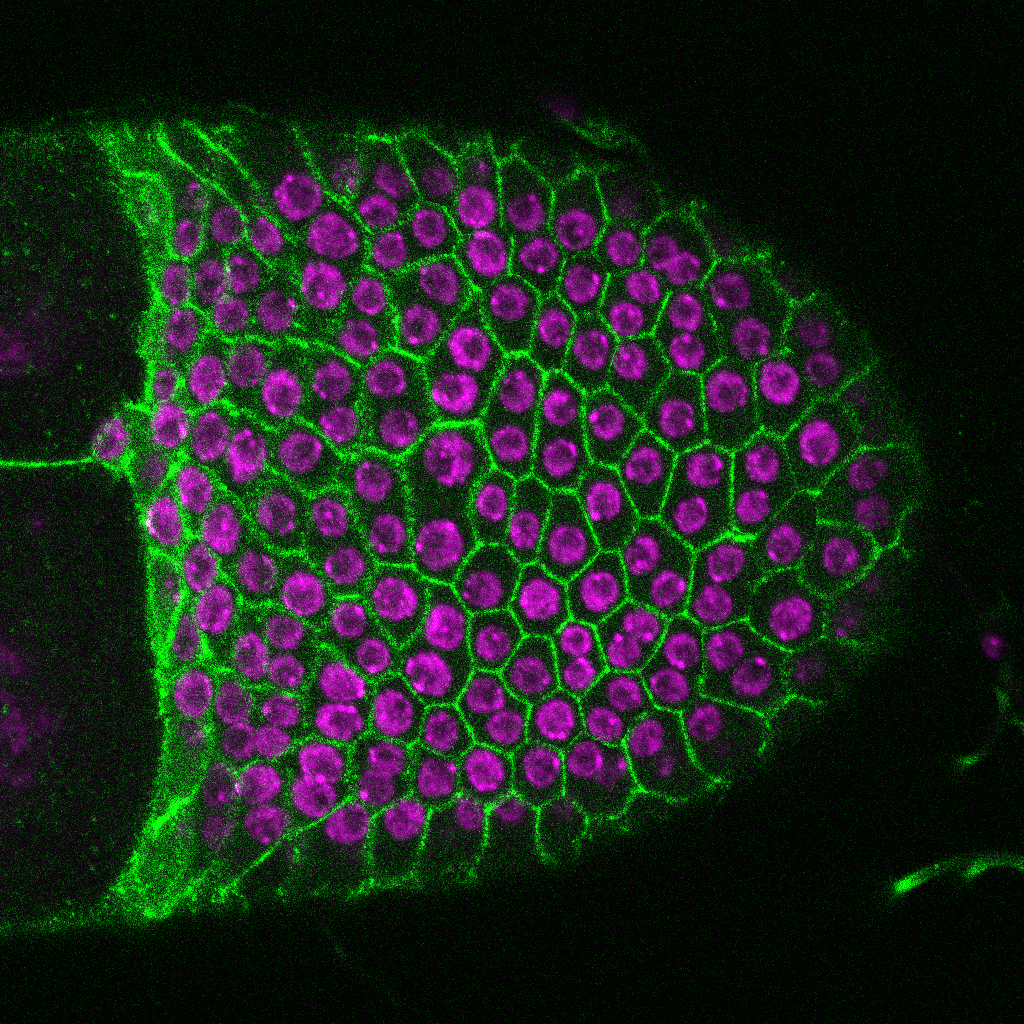

Supplement: Supplementary file 15 — Source data Fig. 2 [file 44319_2024_319_MOESM15_ESM.zip › Figure 2/2C/AniRNAi.tif]

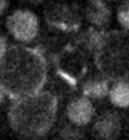

Supplement: Supplementary file 15 — Source data Fig. 2 [file 44319_2024_319_MOESM15_ESM.zip › Figure 2/2D/Control_endofconstriction.tif]

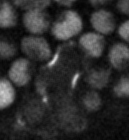

Supplement: Supplementary file 15 — Source data Fig. 2 [file 44319_2024_319_MOESM15_ESM.zip › Figure 2/2D/DysMI025024_ringconstriction.tif]

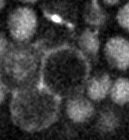

Supplement: Supplementary file 15 — Source data Fig. 2 [file 44319_2024_319_MOESM15_ESM.zip › Figure 2/2D/Control_metaphase.tif]

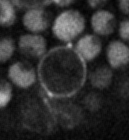

Supplement: Supplementary file 15 — Source data Fig. 2 [file 44319_2024_319_MOESM15_ESM.zip › Figure 2/2D/DysMI025024_metaphase.tif]

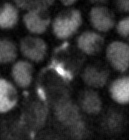

Supplement: Supplementary file 15 — Source data Fig. 2 [file 44319_2024_319_MOESM15_ESM.zip › Figure 2/2D/DysMI024025_endofconstriction.tif]

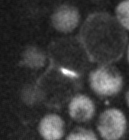

Supplement: Supplementary file 15 — Source data Fig. 2 [file 44319_2024_319_MOESM15_ESM.zip › Figure 2/2D/DysE17_endofconstriction.tif]

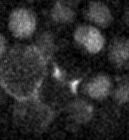

Supplement: Supplementary file 15 — Source data Fig. 2 [file 44319_2024_319_MOESM15_ESM.zip › Figure 2/2D/Control_ringconstriction.tif]

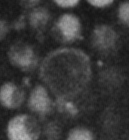

Supplement: Supplementary file 15 — Source data Fig. 2 [file 44319_2024_319_MOESM15_ESM.zip › Figure 2/2D/DysE17_metaphase.tif]

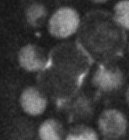

Supplement: Supplementary file 15 — Source data Fig. 2 [file 44319_2024_319_MOESM15_ESM.zip › Figure 2/2D/DysE17_ringconstriction.tif]

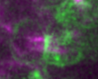

Supplement: Supplementary file 16 — Source data Fig. 3 [file 44319_2024_319_MOESM16_ESM.zip › Figure 3/3E/Dys(green)_Tubulin(magenta)_t=2.tif]

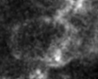

Supplement: Supplementary file 16 — Source data Fig. 3 [file 44319_2024_319_MOESM16_ESM.zip › Figure 3/3E/Dys_t=0.tif]

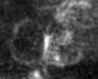

Supplement: Supplementary file 16 — Source data Fig. 3 [file 44319_2024_319_MOESM16_ESM.zip › Figure 3/3E/Dys_t=2.tif]

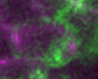

Supplement: Supplementary file 16 — Source data Fig. 3 [file 44319_2024_319_MOESM16_ESM.zip › Figure 3/3E/Dys(green)_Tubulin(magenta)_t=0.tif]

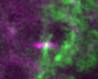

Supplement: Supplementary file 16 — Source data Fig. 3 [file 44319_2024_319_MOESM16_ESM.zip › Figure 3/3E/Dys(green)_Tubulin(magenta)_t=10.tif]

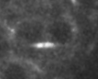

Supplement: Supplementary file 16 — Source data Fig. 3 [file 44319_2024_319_MOESM16_ESM.zip › Figure 3/3E/Tubulin_t=10.tif]

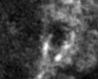

Supplement: Supplementary file 16 — Source data Fig. 3 [file 44319_2024_319_MOESM16_ESM.zip › Figure 3/3E/Dys_t=10.tif]

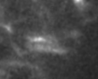

Supplement: Supplementary file 16 — Source data Fig. 3 [file 44319_2024_319_MOESM16_ESM.zip › Figure 3/3E/Tubulin_t=2.tif]

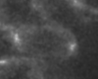

Supplement: Supplementary file 16 — Source data Fig. 3 [file 44319_2024_319_MOESM16_ESM.zip › Figure 3/3E/Tubulin_t=0.tif]

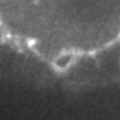

Supplement: Supplementary file 16 — Source data Fig. 3 [file 44319_2024_319_MOESM16_ESM.zip › Figure 3/3B/SqhmKate2_t=2.tif]

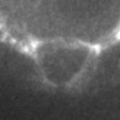

Supplement: Supplementary file 16 — Source data Fig. 3 [file 44319_2024_319_MOESM16_ESM.zip › Figure 3/3B/SqhmKate2_t=0.tif]

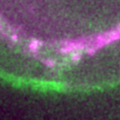

Supplement: Supplementary file 16 — Source data Fig. 3 [file 44319_2024_319_MOESM16_ESM.zip › Figure 3/3B/Dg(green)_SqhmKate2(magenta)_t=5.tif]

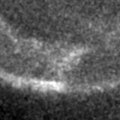

Supplement: Supplementary file 16 — Source data Fig. 3 [file 44319_2024_319_MOESM16_ESM.zip › Figure 3/3B/Dg_t=5.tif]

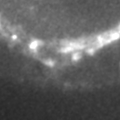

Supplement: Supplementary file 16 — Source data Fig. 3 [file 44319_2024_319_MOESM16_ESM.zip › Figure 3/3B/SqhmKate2_t=5.tif]

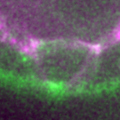

Supplement: Supplementary file 16 — Source data Fig. 3 [file 44319_2024_319_MOESM16_ESM.zip › Figure 3/3B/Dg(green)_SqhmKate2(magenta)_t=0.tif]

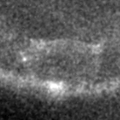

Supplement: Supplementary file 16 — Source data Fig. 3 [file 44319_2024_319_MOESM16_ESM.zip › Figure 3/3B/Dg_t=0.tif]

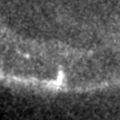

Supplement: Supplementary file 16 — Source data Fig. 3 [file 44319_2024_319_MOESM16_ESM.zip › Figure 3/3B/Dg_t=2.tif]

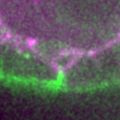

Supplement: Supplementary file 16 — Source data Fig. 3 [file 44319_2024_319_MOESM16_ESM.zip › Figure 3/3B/Dg(green)_SqhmKate2(magenta)_t=2.tif]

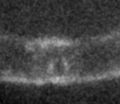

Supplement: Supplementary file 16 — Source data Fig. 3 [file 44319_2024_319_MOESM16_ESM.zip › Figure 3/3C/Dys_t=6..tif]

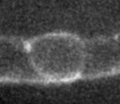

Supplement: Supplementary file 16 — Source data Fig. 3 [file 44319_2024_319_MOESM16_ESM.zip › Figure 3/3C/Dys_t=0.tif]

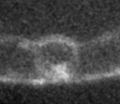

Supplement: Supplementary file 16 — Source data Fig. 3 [file 44319_2024_319_MOESM16_ESM.zip › Figure 3/3C/Dys_t=2.tif]

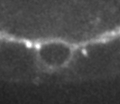

Supplement: Supplementary file 16 — Source data Fig. 3 [file 44319_2024_319_MOESM16_ESM.zip › Figure 3/3C/SqhmKate2_t=2.tif]

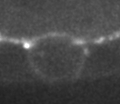

Supplement: Supplementary file 16 — Source data Fig. 3 [file 44319_2024_319_MOESM16_ESM.zip › Figure 3/3C/SqhmKate2_t=0.tif]

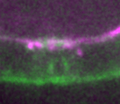

Supplement: Supplementary file 16 — Source data Fig. 3 [file 44319_2024_319_MOESM16_ESM.zip › Figure 3/3C/Dys(green)_SqhmKate2(magenta)_t=6.tif]

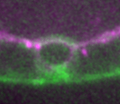

Supplement: Supplementary file 16 — Source data Fig. 3 [file 44319_2024_319_MOESM16_ESM.zip › Figure 3/3C/Dys(green)_SqhmKate2(magenta)_t=2.tif]

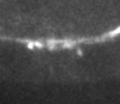

Supplement: Supplementary file 16 — Source data Fig. 3 [file 44319_2024_319_MOESM16_ESM.zip › Figure 3/3C/SqhmKate2_t=6.tif]

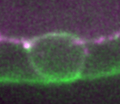

Supplement: Supplementary file 16 — Source data Fig. 3 [file 44319_2024_319_MOESM16_ESM.zip › Figure 3/3C/Dys(green)_SqhmKate2(magenta)_t=0.tif]

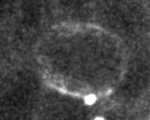

Supplement: Supplementary file 16 — Source data Fig. 3 [file 44319_2024_319_MOESM16_ESM.zip › Figure 3/3D/SqhmKate2(magenta)_timeprojection_30secperframe..tif]

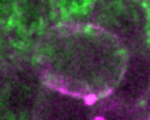

Supplement: Supplementary file 16 — Source data Fig. 3 [file 44319_2024_319_MOESM16_ESM.zip › Figure 3/3D/Dys(green)_SqhmKate2(magenta)_timeprojection_30secperframe.tif]

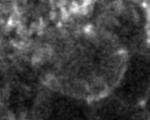

Supplement: Supplementary file 16 — Source data Fig. 3 [file 44319_2024_319_MOESM16_ESM.zip › Figure 3/3D/Dys(green)_timeprojection_30secperframe.tif]

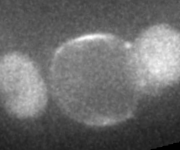

Supplement: Supplementary file 16 — Source data Fig. 3 [file 44319_2024_319_MOESM16_ESM.zip › Figure 3/3A/Anillin(magenta)_timeprojection_30secperframe.tif]

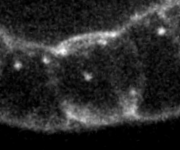

Supplement: Supplementary file 16 — Source data Fig. 3 [file 44319_2024_319_MOESM16_ESM.zip › Figure 3/3A/Dg(green)_timeprojection_30secperframe.tif]

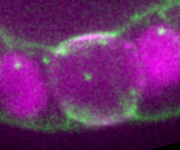

Supplement: Supplementary file 16 — Source data Fig. 3 [file 44319_2024_319_MOESM16_ESM.zip › Figure 3/3A/Dg(green)_Anillin(magenta)_timeprojection_30secperframe.tif]

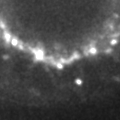

Supplement: Supplementary file 17 — Source data Fig. 4 [file 44319_2024_319_MOESM17_ESM.zip › Figure 4/4B/Dyslong_SqhmKate2(magenta)_t=7.5.tif]

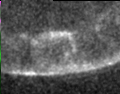

Supplement: Supplementary file 17 — Source data Fig. 4 [file 44319_2024_319_MOESM17_ESM.zip › Figure 4/4B/Dysshort(green)_t=7.5.tif]

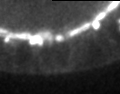

Supplement: Supplementary file 17 — Source data Fig. 4 [file 44319_2024_319_MOESM17_ESM.zip › Figure 4/4B/Dysshort_SqhmKate2(magenta)_t=7.5.tif]

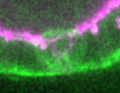

Supplement: Supplementary file 17 — Source data Fig. 4 [file 44319_2024_319_MOESM17_ESM.zip › Figure 4/4B/Dysshort(green)_SqhmKate2(magenta)_t=2.tif]

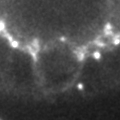

Supplement: Supplementary file 17 — Source data Fig. 4 [file 44319_2024_319_MOESM17_ESM.zip › Figure 4/4B/Dyslong_SqhmKate2(magenta)_t=0.tif]

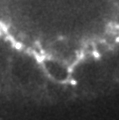

Supplement: Supplementary file 17 — Source data Fig. 4 [file 44319_2024_319_MOESM17_ESM.zip › Figure 4/4B/Dyslong_SqhmKate2(magenta)_t=1.tif]

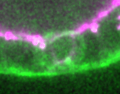

Supplement: Supplementary file 17 — Source data Fig. 4 [file 44319_2024_319_MOESM17_ESM.zip › Figure 4/4B/Dysshort(green)_SqhmKate2(magenta)_t=1.tif]

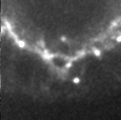

Supplement: Supplementary file 17 — Source data Fig. 4 [file 44319_2024_319_MOESM17_ESM.zip › Figure 4/4B/Dyslong_SqhmKate2(magenta)_t=2.tif]

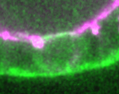

Supplement: Supplementary file 17 — Source data Fig. 4 [file 44319_2024_319_MOESM17_ESM.zip › Figure 4/4B/Dysshort(green)_SqhmKate2(magenta)_t=0.tif]

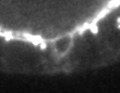

Supplement: Supplementary file 17 — Source data Fig. 4 [file 44319_2024_319_MOESM17_ESM.zip › Figure 4/4B/Dysshort_SqhmKate2(magenta)_t=2.tif]

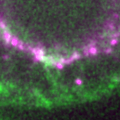

Supplement: Supplementary file 17 — Source data Fig. 4 [file 44319_2024_319_MOESM17_ESM.zip › Figure 4/4B/Dyslong(green)_SqhmKate2(magenta)_t=7.5.tif]

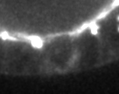

Supplement: Supplementary file 17 — Source data Fig. 4 [file 44319_2024_319_MOESM17_ESM.zip › Figure 4/4B/Dysshort_SqhmKate2(magenta)_t=0.tif]

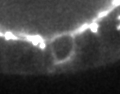

Supplement: Supplementary file 17 — Source data Fig. 4 [file 44319_2024_319_MOESM17_ESM.zip › Figure 4/4B/Dysshort_SqhmKate2(magenta)_t=1.tif]

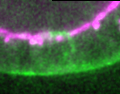

Supplement: Supplementary file 17 — Source data Fig. 4 [file 44319_2024_319_MOESM17_ESM.zip › Figure 4/4B/Dysshort(green)_SqhmKate2(magenta)_t=7.5.tif]

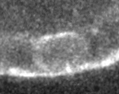

Supplement: Supplementary file 17 — Source data Fig. 4 [file 44319_2024_319_MOESM17_ESM.zip › Figure 4/4B/Dysshort(green)_t=0.tif]

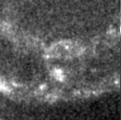

Supplement: Supplementary file 17 — Source data Fig. 4 [file 44319_2024_319_MOESM17_ESM.zip › Figure 4/4B/Dyslong(green)_t=2.tif]

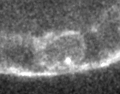

Supplement: Supplementary file 17 — Source data Fig. 4 [file 44319_2024_319_MOESM17_ESM.zip › Figure 4/4B/Dysshort(green)_t=1.tif]

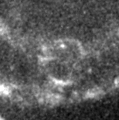

Supplement: Supplementary file 17 — Source data Fig. 4 [file 44319_2024_319_MOESM17_ESM.zip › Figure 4/4B/Dyslong(green)_t=1.tif]

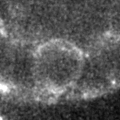

Supplement: Supplementary file 17 — Source data Fig. 4 [file 44319_2024_319_MOESM17_ESM.zip › Figure 4/4B/Dyslong(green)_t=0.tif]

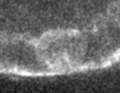

Supplement: Supplementary file 17 — Source data Fig. 4 [file 44319_2024_319_MOESM17_ESM.zip › Figure 4/4B/Dysshort(green)_t=2.tif]

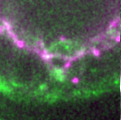

Supplement: Supplementary file 17 — Source data Fig. 4 [file 44319_2024_319_MOESM17_ESM.zip › Figure 4/4B/Dyslong(green)_SqhmKate2(magenta)_t=2.tif]

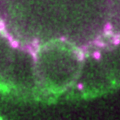

Supplement: Supplementary file 17 — Source data Fig. 4 [file 44319_2024_319_MOESM17_ESM.zip › Figure 4/4B/Dyslong(green)_SqhmKate2(magenta)_t=0.tif]

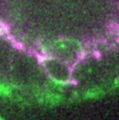

Supplement: Supplementary file 17 — Source data Fig. 4 [file 44319_2024_319_MOESM17_ESM.zip › Figure 4/4B/Dyslong(green)_SqhmKate2(magenta)_t=1.tif]

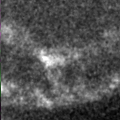

Supplement: Supplementary file 17 — Source data Fig. 4 [file 44319_2024_319_MOESM17_ESM.zip › Figure 4/4B/Dyslong(green)_t=7.5.tif]

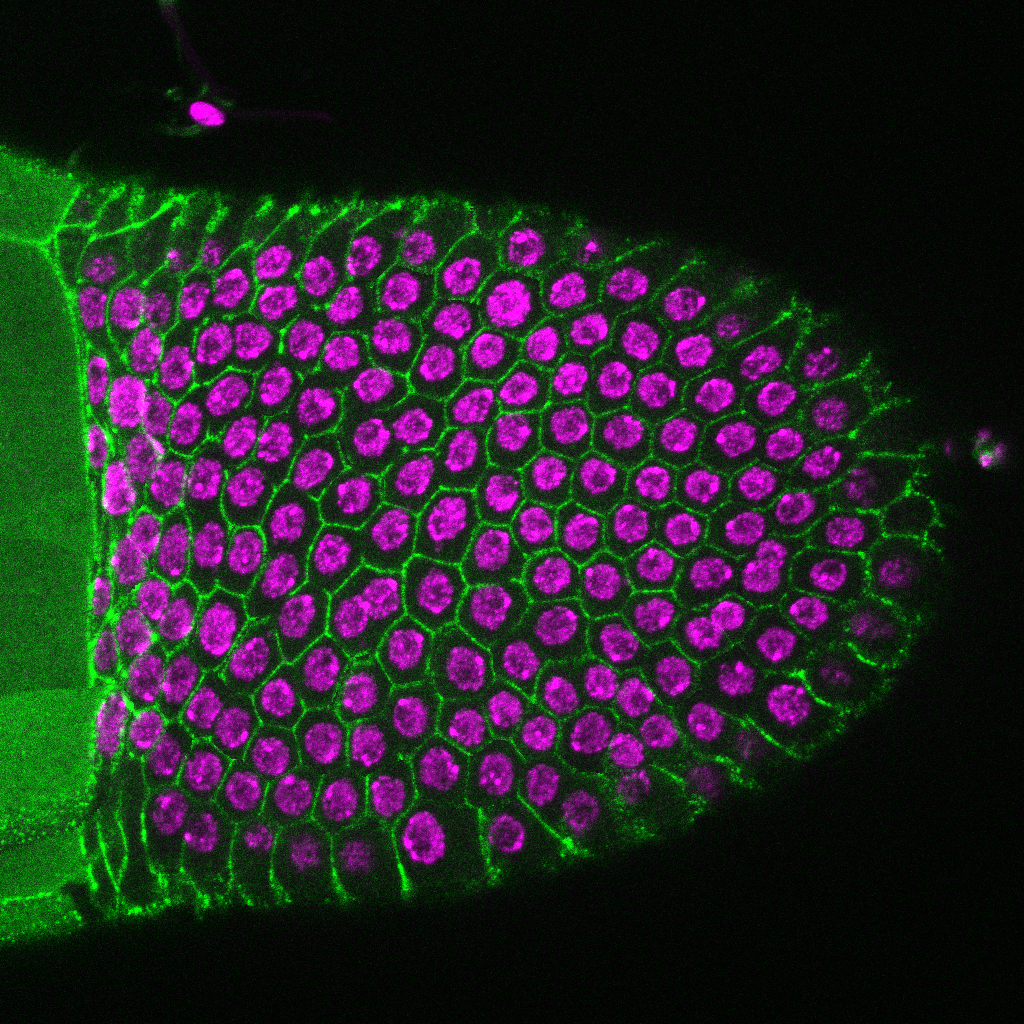

Supplement: Supplementary file 17 — Source data Fig. 4 [file 44319_2024_319_MOESM17_ESM.zip › Figure 4/4D/Dyslong181_EcadGFP_DAPI.tif]

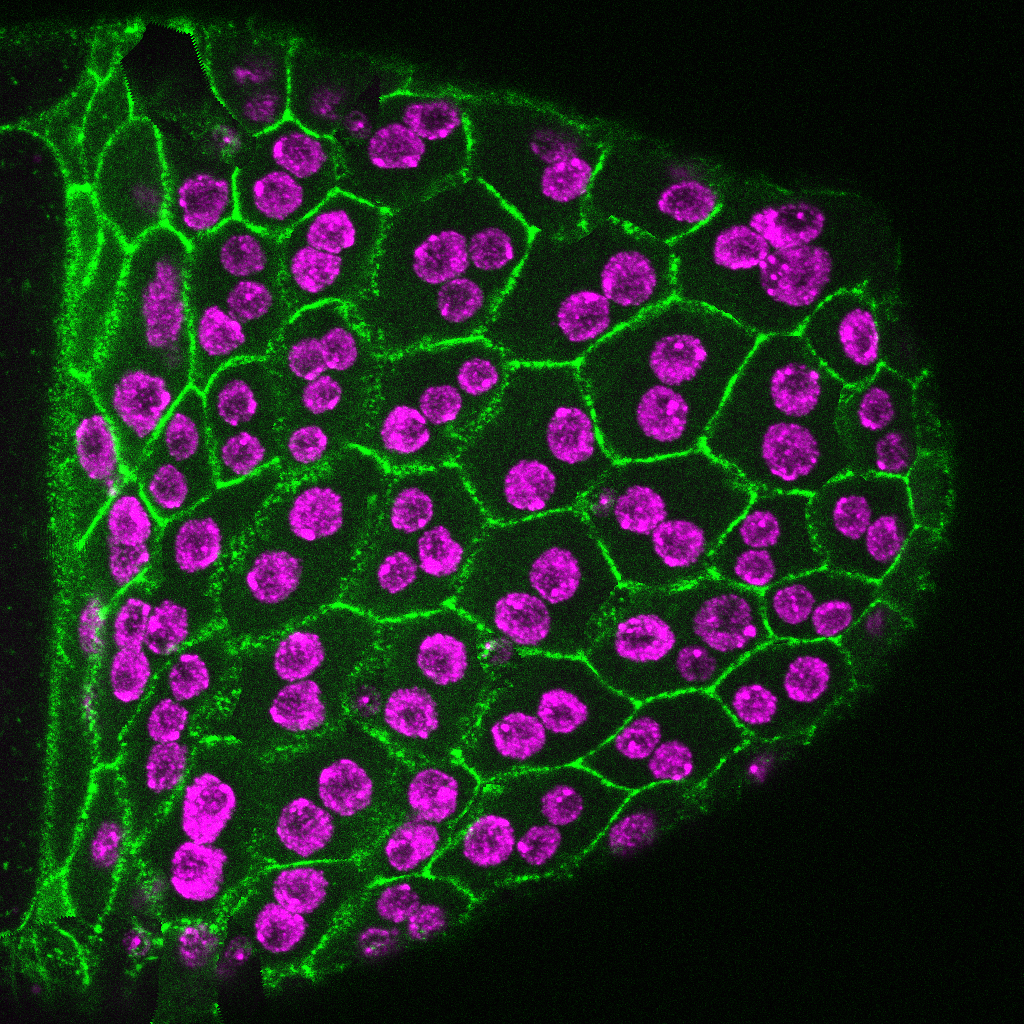

Supplement: Supplementary file 17 — Source data Fig. 4 [file 44319_2024_319_MOESM17_ESM.zip › Figure 4/4D/Dyslong181+AniRNAi._EcadGFP_DAPI.tif]

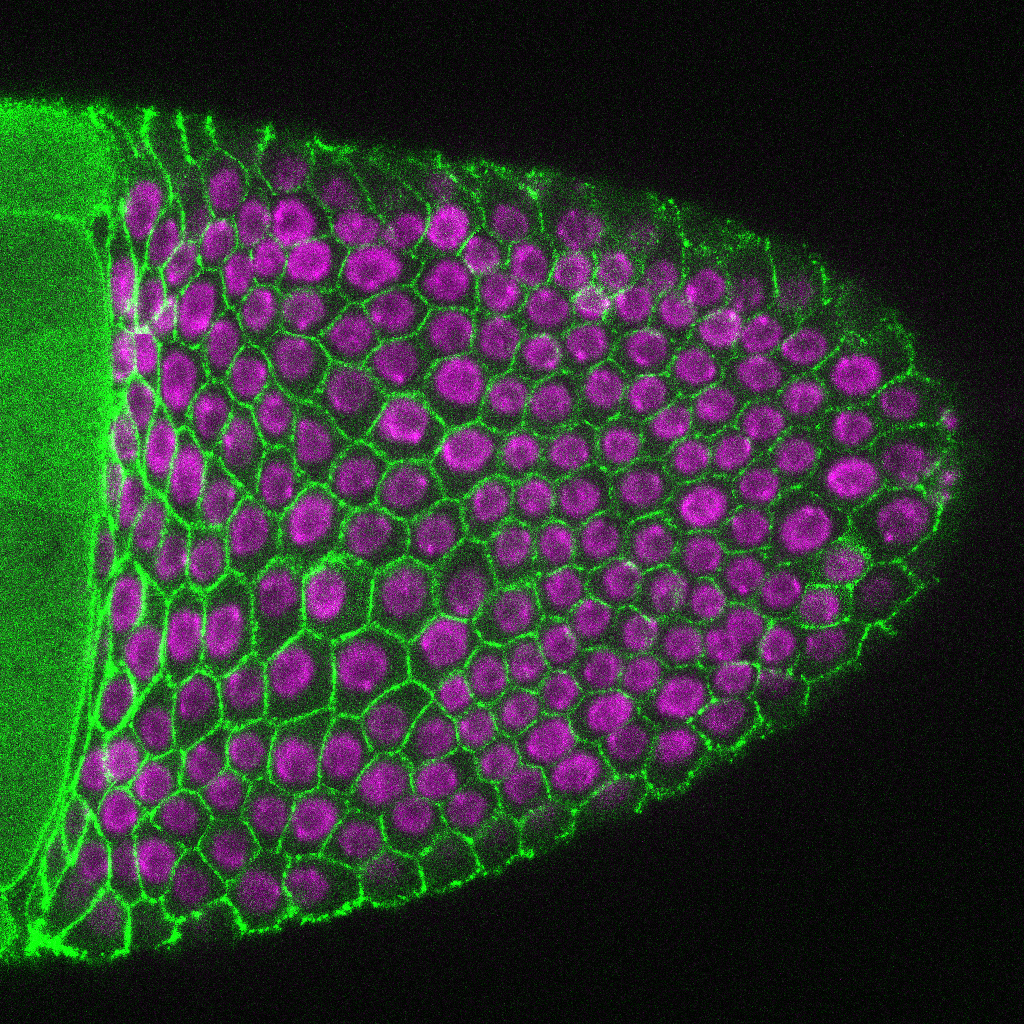

Supplement: Supplementary file 17 — Source data Fig. 4 [file 44319_2024_319_MOESM17_ESM.zip › Figure 4/4D/DysRE225_EcadGFP_DAPI.tif]

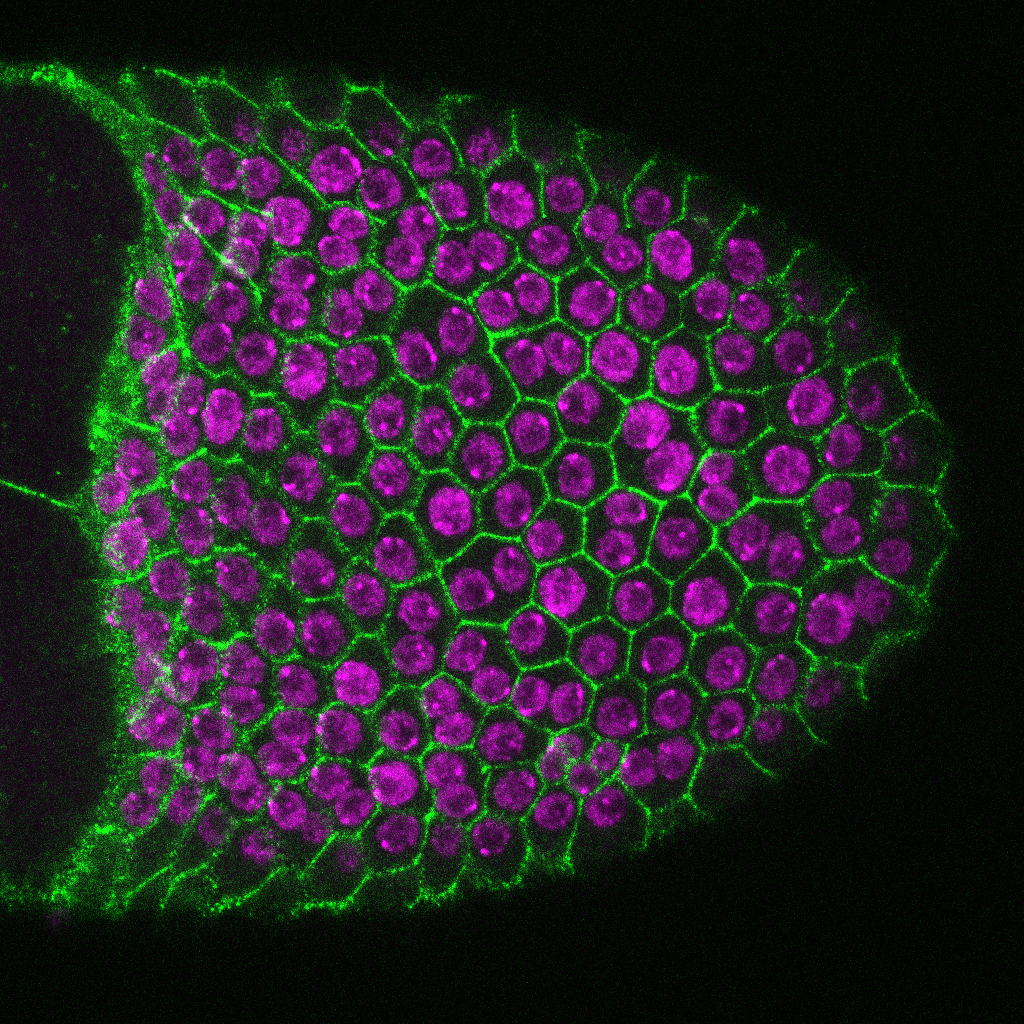

Supplement: Supplementary file 17 — Source data Fig. 4 [file 44319_2024_319_MOESM17_ESM.zip › Figure 4/4D/AniRNAi_EcadGFP_DAPI.tif]

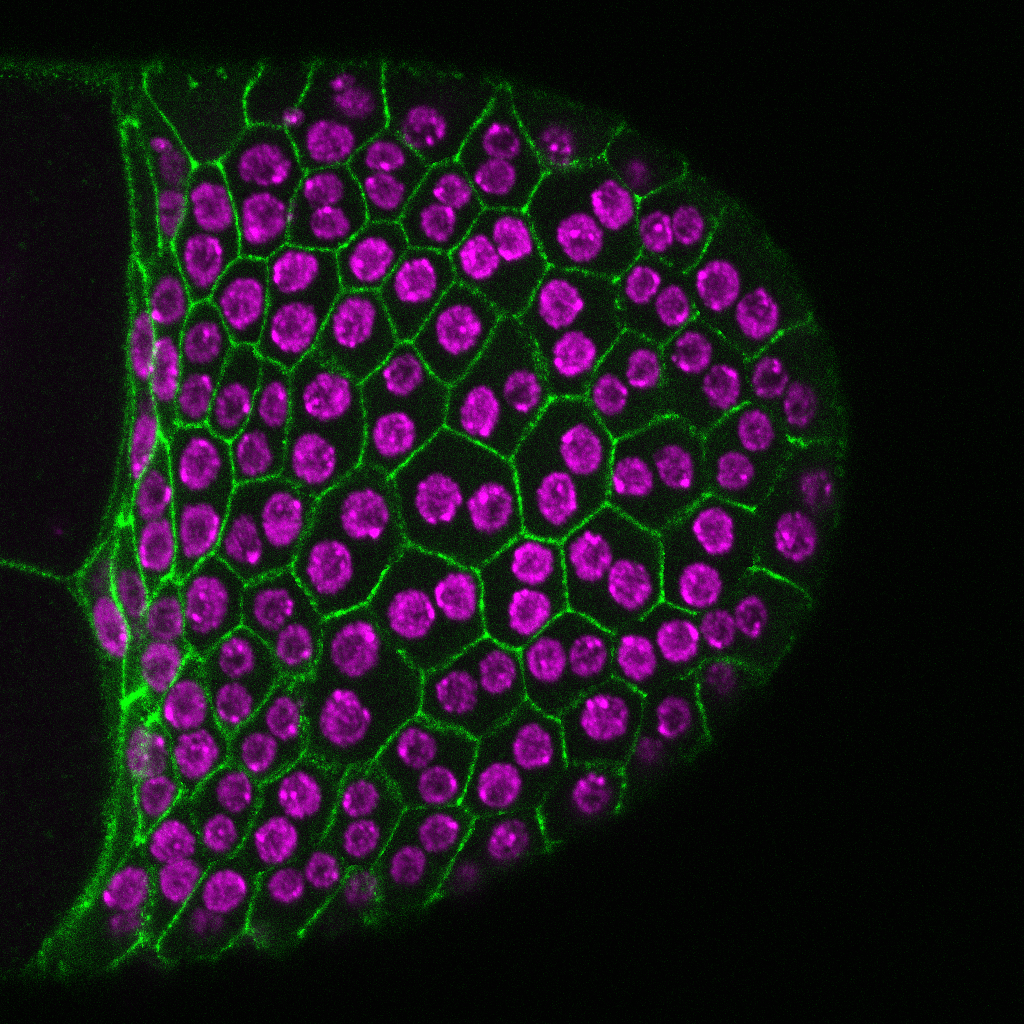

Supplement: Supplementary file 17 — Source data Fig. 4 [file 44319_2024_319_MOESM17_ESM.zip › Figure 4/4D/DysRE225+AniRNAi_EcadGFP_DAPI.tif]

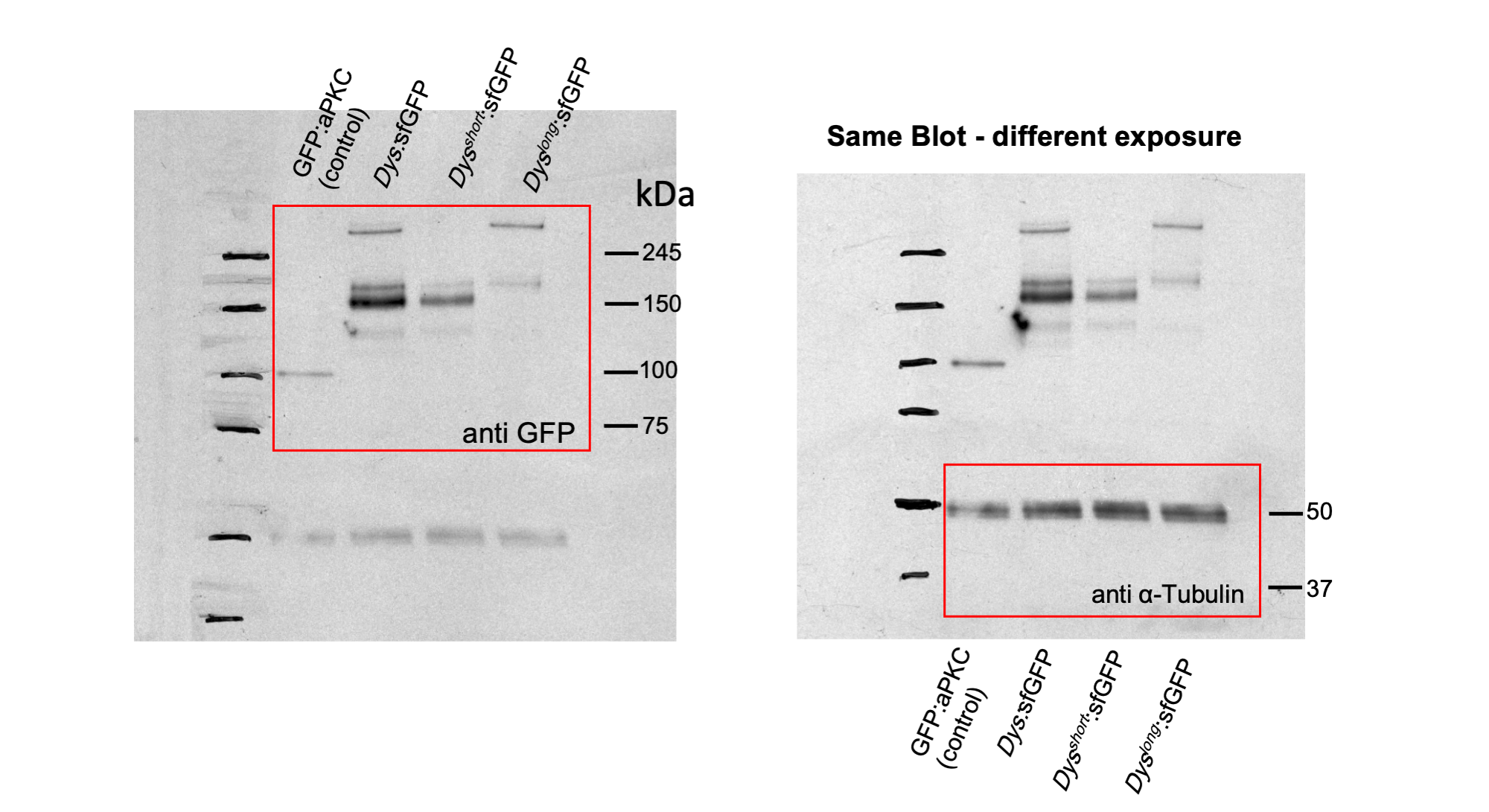

Supplement: Supplementary file 17 — Source data Fig. 4 [file 44319_2024_319_MOESM17_ESM.zip › Figure 4/4A/4A.tiff]

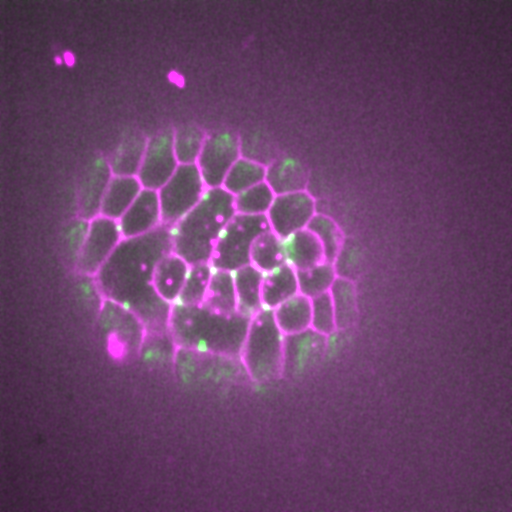

Supplement: Supplementary file 18 — Source data Fig. 5 [file 44319_2024_319_MOESM18_ESM.zip › Figure 5/5A/AniRNAi_CellMask(magenta)_ZipGFP(green).tif]

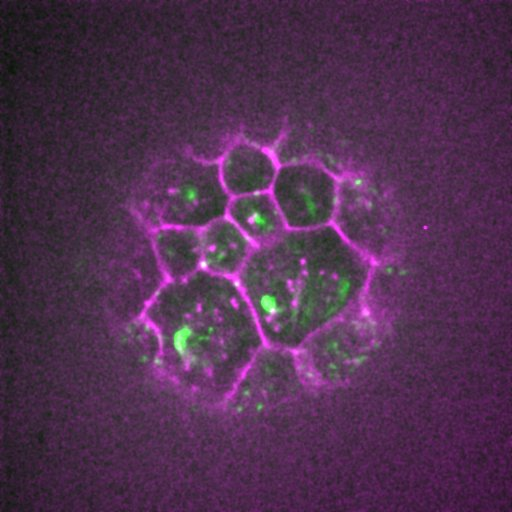

Supplement: Supplementary file 18 — Source data Fig. 5 [file 44319_2024_319_MOESM18_ESM.zip › Figure 5/5A/AniRNAi_DysE17_CellMask(magenta)_ZipGFP(green).tif]

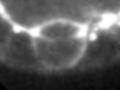

Supplement: Supplementary file 18 — Source data Fig. 5 [file 44319_2024_319_MOESM18_ESM.zip › Figure 5/5E/Dg_SqhmKate2_timeprojection_30secperframe.tif]

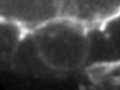

Supplement: Supplementary file 18 — Source data Fig. 5 [file 44319_2024_319_MOESM18_ESM.zip › Figure 5/5E/Dys_SqhmKate2_timeprojection_30secperframe.tif]

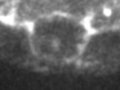

Supplement: Supplementary file 18 — Source data Fig. 5 [file 44319_2024_319_MOESM18_ESM.zip › Figure 5/5E/Control_SqhmKate2_timeprojection_30secperframe.tif]

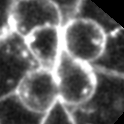

Supplement: Supplementary file 18 — Source data Fig. 5 [file 44319_2024_319_MOESM18_ESM.zip › Figure 5/5B/AnillinRNAiDysE17_CellMask(magenta)_t=5.tif]

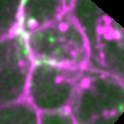

Supplement: Supplementary file 18 — Source data Fig. 5 [file 44319_2024_319_MOESM18_ESM.zip › Figure 5/5B/AnillinRNAiDysE17_CellMask(magenta)_Zip(green)_t=38.tif]

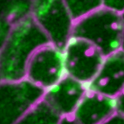

Supplement: Supplementary file 18 — Source data Fig. 5 [file 44319_2024_319_MOESM18_ESM.zip › Figure 5/5B/Control_CellMask(magenta)_Zip(green)_t=2.5.tif]

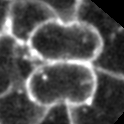

Supplement: Supplementary file 18 — Source data Fig. 5 [file 44319_2024_319_MOESM18_ESM.zip › Figure 5/5B/AnillinRNAiDysE17_CellMask(magenta)_t=0.tif]

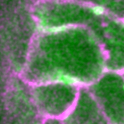

Supplement: Supplementary file 18 — Source data Fig. 5 [file 44319_2024_319_MOESM18_ESM.zip › Figure 5/5B/AnillinRNAi_CellMask(magenta)_Zip(green)_t=0.tif]

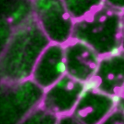

Supplement: Supplementary file 18 — Source data Fig. 5 [file 44319_2024_319_MOESM18_ESM.zip › Figure 5/5B/Control_CellMask(magenta)_Zip(green)_t=31.tif]

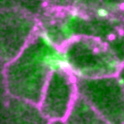

Supplement: Supplementary file 18 — Source data Fig. 5 [file 44319_2024_319_MOESM18_ESM.zip › Figure 5/5B/AnillinRNAi_CellMask(magenta)_Zip(green)_t=15.tif]

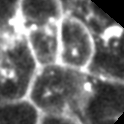

Supplement: Supplementary file 18 — Source data Fig. 5 [file 44319_2024_319_MOESM18_ESM.zip › Figure 5/5B/AnillinRNAiDysE17_CellMask(magenta)_t=25.tif]

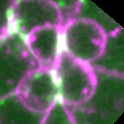

Supplement: Supplementary file 18 — Source data Fig. 5 [file 44319_2024_319_MOESM18_ESM.zip › Figure 5/5B/AnillinRNAiDysE17_CellMask(magenta)_Zip(green)_t=5.tif]

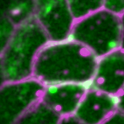

Supplement: Supplementary file 18 — Source data Fig. 5 [file 44319_2024_319_MOESM18_ESM.zip › Figure 5/5B/Control_CellMask(magenta)_Zip(green)_t=0.tif]

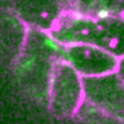

Supplement: Supplementary file 18 — Source data Fig. 5 [file 44319_2024_319_MOESM18_ESM.zip › Figure 5/5B/AnillinRNAi_CellMask(magenta)_Zip(green)_t=25.tif]

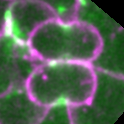

Supplement: Supplementary file 18 — Source data Fig. 5 [file 44319_2024_319_MOESM18_ESM.zip › Figure 5/5B/AnillinRNAiDysE17_CellMask(magenta)_Zip(green)_t=0.tif]

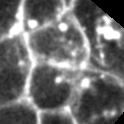

Supplement: Supplementary file 18 — Source data Fig. 5 [file 44319_2024_319_MOESM18_ESM.zip › Figure 5/5B/AnillinRNAiDysE17_CellMask(magenta)_t=38.tif]

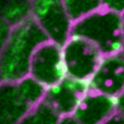

Supplement: Supplementary file 18 — Source data Fig. 5 [file 44319_2024_319_MOESM18_ESM.zip › Figure 5/5B/Control_CellMask(magenta)_Zip(green)_t=5.tif]

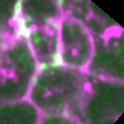

Supplement: Supplementary file 18 — Source data Fig. 5 [file 44319_2024_319_MOESM18_ESM.zip › Figure 5/5B/AnillinRNAiDysE17_CellMask(magenta)_Zip(green)_t=25.tif]

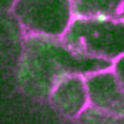

Supplement: Supplementary file 18 — Source data Fig. 5 [file 44319_2024_319_MOESM18_ESM.zip › Figure 5/5B/AnillinRNAi_CellMask(magenta)_Zip(green)_t=40.tif]
