# Supplementary material for: The Dystrophin-Dystroglycan complex ensures cytokinesis efficiency in Drosophila epithelia
Source: EMBO Rep. 2024 Nov 15;26(2):307–28. doi: 10.1038/s44319-024-00319-y (PMC11772804; doi:10.1038/s44319-024-00319-y)
Supplement: Supplementary file 19 — Expanded View Figures [file 44319_2024_319_MOESM19_ESM.pdf]

Expanded View Figures

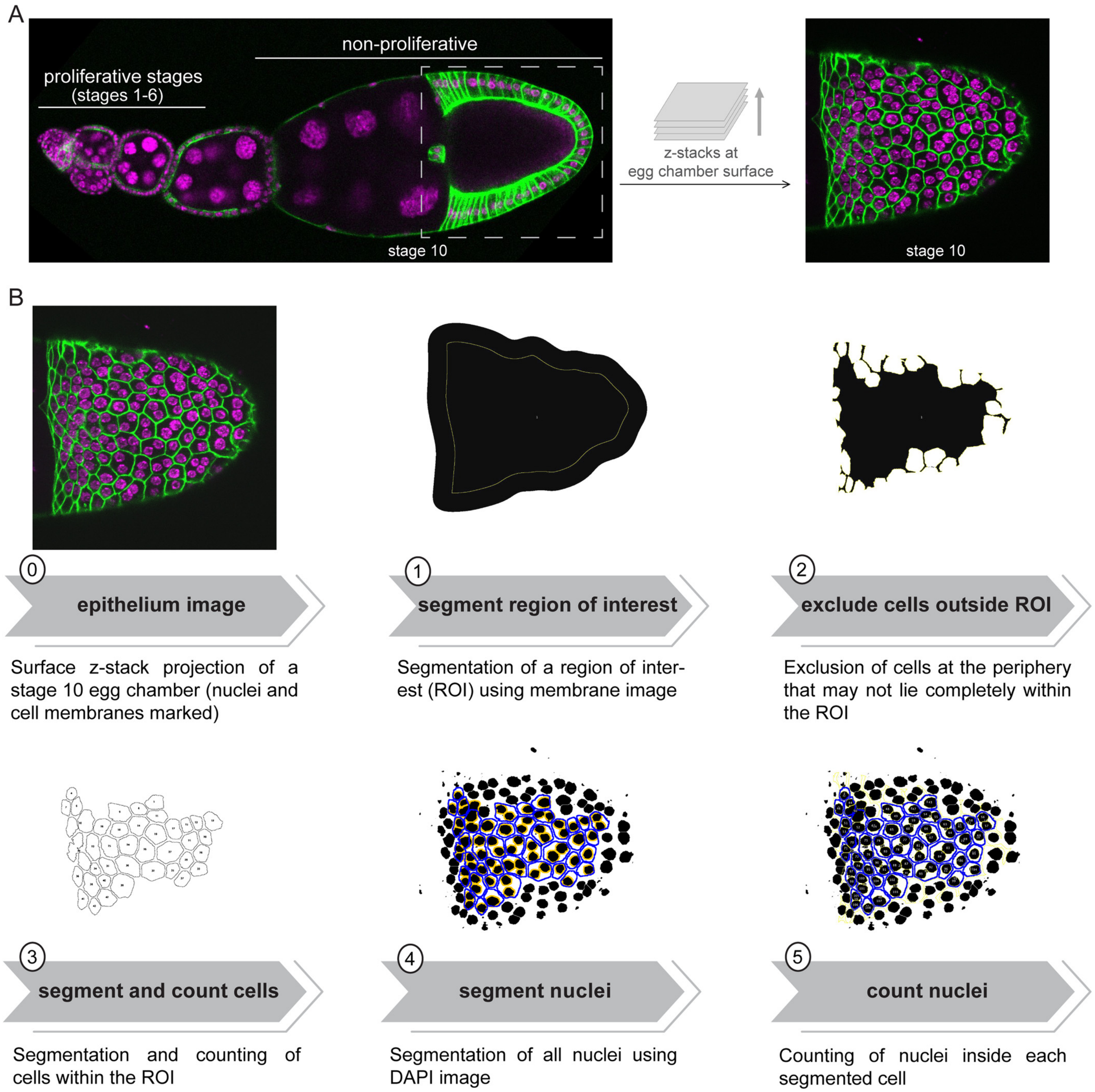

Figure EV1. Pipeline for the quantification of cytokinesis defects in the follicular epithelium of stage 10 egg chambers.

(A) Surface z-projections of non-proliferative stage 10 egg chambers were used to quantify the cytokinesis defects from the in vivo genetic modifier screen (nuclei marked with DAPI (magenta) and cell membranes endogenously expressing Myr:GFP (green)). At this stage of oogenesis, follicle cells are no longer undergoing mitosis, allowing us to directly correlate multinucleation with cytokinesis failure, and are larger than at younger stages, facilitating the automated segmentation of nuclei and cell membranes. **(B)** A macro for FIJI was developed for the automated segmentation and counting of nuclei and cells in two-channel images of the follicular epithelium (nuclei in magenta and cell membranes in green). Note that this was restricted to the central area of the egg chamber, to avoid misleading quantifications from nuclei/cells at the periphery of the egg chambers. After running the macro, a manual validation of the segmentation of both nuclei and cells was performed for all images. The obtained values were used to calculate the Multinucleation Ratio, as explained in detail in the Methods section.

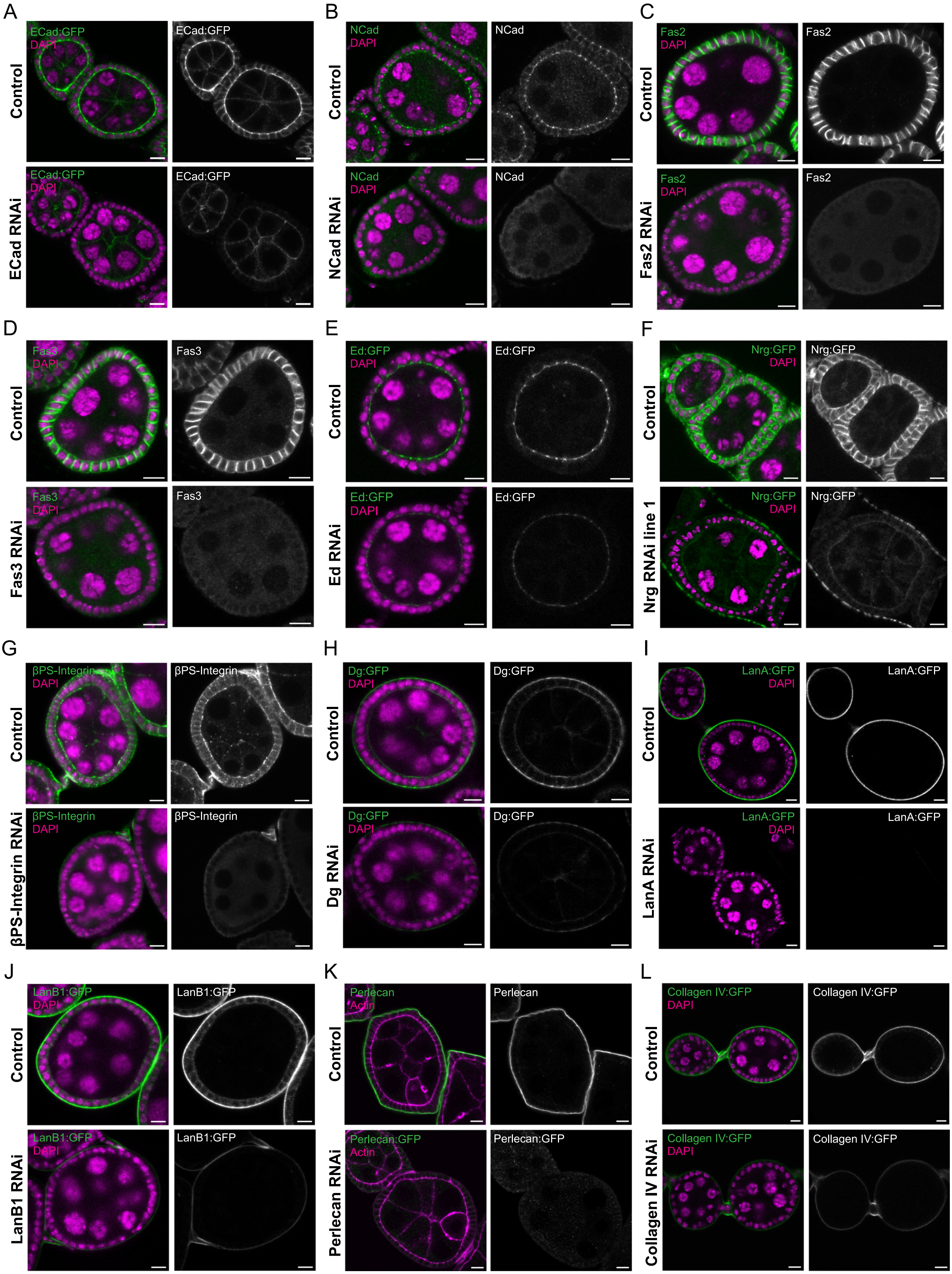
A
Control
ECad:GFP
DAPI
ECad:GFP
ECad RNAi
ECad:GFP
DAPI
ECad:GFP
B
Control
NCad
DAPI
NCad
NCad RNAi
NCad
DAPI
NCad
C
Control
Fas2
DAPI
Fas2
Fas2 RNAi
Fas2
DAPI
Fas2
D
Control
Fas3
DAPI
Fas3
Fas3 RNAi
Fas3
DAPI
Fas3
E
Control
Ed:GFP
DAPI
Ed:GFP
Ed RNAi
Ed:GFP
DAPI
Ed:GFP
F
Control
Nrg:GFP
DAPI
Nrg:GFP
Nrg RNAi line 1
Nrg:GFP
DAPI
Nrg:GFP
G
Control
βPS-Integrin
DAPI
βPS-Integrin
βPS-Integrin RNAi
βPS-Integrin
DAPI
βPS-Integrin
H
Control
Dg:GFP
DAPI
Dg:GFP
Dg RNAi
Dg:GFP
DAPI
Dg:GFP
I
Control
LanA:GFP
DAPI
LanA:GFP
LanA RNAi
LanA:GFP
DAPI
LanA:GFP
J
Control
LanB1:GFP
DAPI
LanB1:GFP
LanB1 RNAi
LanB1:GFP
DAPI
LanB1:GFP
K
Control
Perlecan
Actin
Perlecan
Perlecan RNAi
Perlecan:GFP
Actin
Perlecan:GFP
L
Control
Collagen IV:GFP
DAPI
Collagen IV:GFP
Collagen IV RNAi
Collagen IV:GFP
DAPI
Collagen IV:GFP

 © The Author(s)

Figure EV2. Validation of RNAi-mediated depletion of proteins from the genetic modifier screen.

(**A**) Midsagittal images of control and ECad RNAi egg chambers endogenously expressing ECad:GFP (green) and DAPI (magenta). (**B**) Midsagittal images of control and NCad RNAi egg chambers, stained for NCad (green) and DAPI (magenta). (**C**) Midsagittal images of control and Fas2 RNAi egg chambers, stained for Fas2 (green) and DAPI (magenta). (**D**) Midsagittal images of control and Fas3 RNAi egg chambers, stained for Fas3 (green) and DAPI (magenta). (**E**) Midsagittal images of control and Ed RNAi egg chambers endogenously expressing Ed:GFP (green) and stained for DAPI (magenta). (**F**) Midsagittal images of control and Nrg RNAi (line 1) egg chambers endogenously expressing Nrg:GFP (green) and stained for DAPI (magenta). (**G**) Midsagittal images of control and βPS-integrin RNAi egg chambers, stained for βPS-integrin (green) and F-actin (magenta). (**H**) Midsagittal images of control and Dg RNAi egg chambers endogenously expressing Dg:GFP (green) and stained for DAPI (magenta). (**I**) Midsagittal images of control and LanA RNAi egg chambers endogenously expressing LanA:GFP (green) and stained for DAPI (magenta). (**J**) Midsagittal images of control and LanB1 RNAi egg chambers endogenously expressing LanB1:GFP (green) and stained for DAPI (magenta). (**K**) Midsagittal images of control and Perlecan RNAi egg chambers, stained for Perlecan (green) and F-actin (magenta). (**L**) Midsagittal images of control and Collagen IV RNAi egg chambers endogenously expressing Collagen IV:GFP (green) and stained for DAPI (magenta). (**A**–**L**) Scale bars: 10 μm.

© The Author(s)

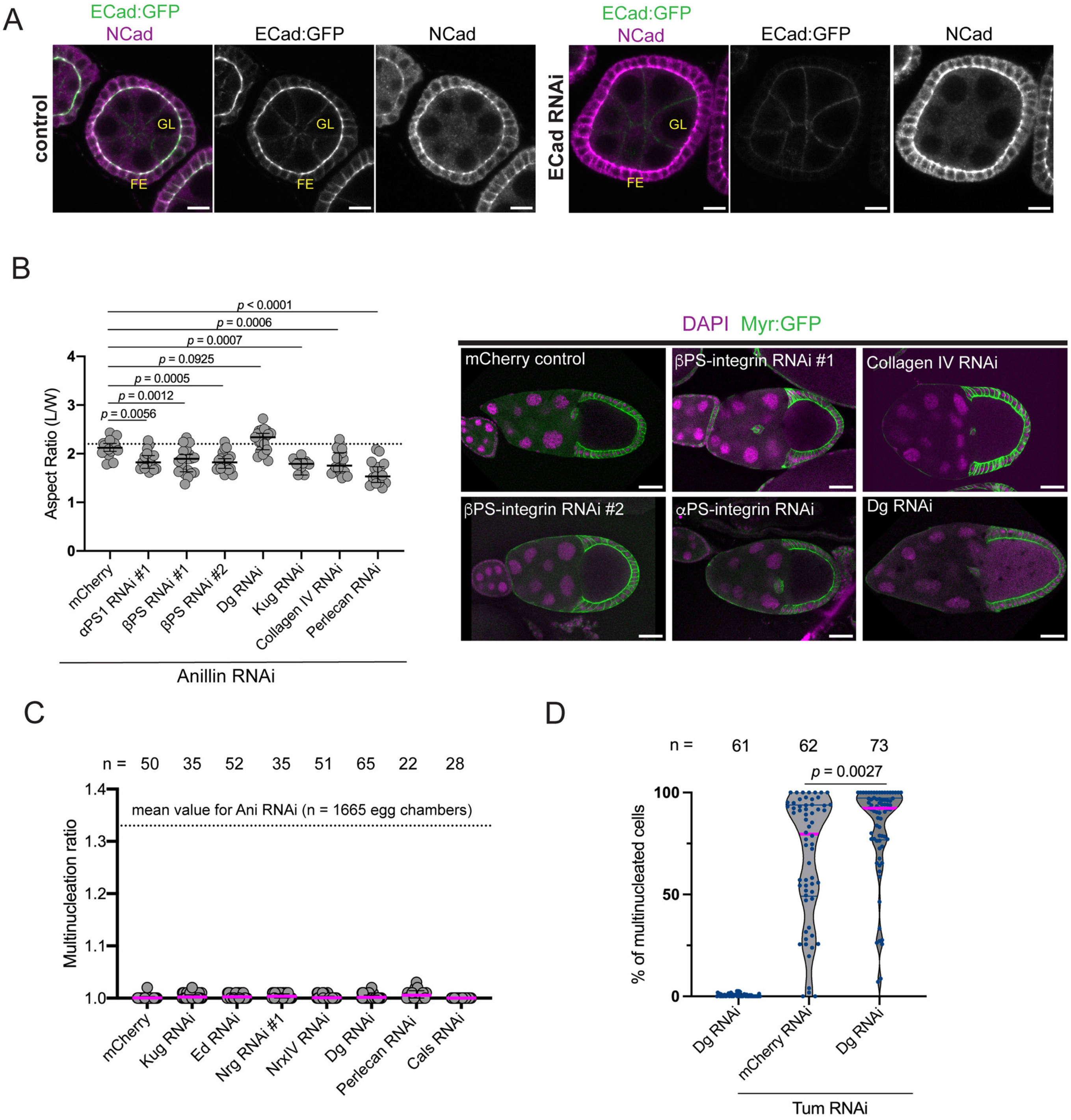
A
ECad:GFP
NCad
ECad:GFP
NCad
control
GL
FE
ECad:GFP
NCad
ECad:GFP
NCad
ECad RNAi
GL
FE
B
p < 0.0001
p = 0.0006
p = 0.0007
p = 0.0925
p = 0.0005
p = 0.0012
p = 0.0056
Aspect Ratio (L/W)
mCherry
αPS1 RNAi #1
βPS RNAi #1
βPS RNAi #2
Dg RNAi
Kug RNAi
Collagen IV RNAi
Perlecan RNAi
Anillin RNAi
DAPI Myr:GFP
mCherry control
βPS-integrin RNAi #1
Collagen IV RNAi
βPS-integrin RNAi #2
αPS-integrin RNAi
Dg RNAi
C
n = 50 35 52 35 51 65 22 28
mean value for Ani RNAi (n = 1665 egg chambers)
Multinucleation ratio
mCherry
Kug RNAi
Ed RNAi
Nrg RNAi #1
NrxIV RNAi
Dg RNAi
Perlecan RNAi
Cals RNAi
D
n = 61 62 73
p = 0.0027
% of multinucleated cells
Dg RNAi
mCherry RNAi
Dg RNAi
Tum RNAi

 © The Author(s)

Figure EV3. Complementary analysis of RNAi-mediated depletion and their effects on cytokinesis.

(**A**) Midsagittal images of control and ECad RNAi egg chambers endogenously expressing ECad:GFP (green) and stained for NCad (magenta). Expression of RNAi for ECad in the follicular epithelium (FE) induces efficient protein depletion. As anticipated, reduction of ECad:GFP fluorescence by ECad RNAi is restricted to the follicular epithelium (FE) and ECad levels are normal in the germline (GL). Scale bar: 10 µm. (**B**) Midsagittal images and measurement of the aspect ratio in stage 10 egg chambers co-depleted for Anillin and the indicated basal proteins, in comparison with control (UAS-mCherry). Aspect ratio was calculated as the ratio between egg chamber length and width, as depicted in the bottom image (DAPI in magenta and cell membrane in green (Myr:GFP)). Each dot represents the aspect ratio of an analyzed egg chamber. The dashed line represents the anticipated aspect ratio value for this egg chamber stage, as calculated in (Jia et al, 2016). Median ± 95 Confidence Interval is shown. *P* value was calculated by an ordinary one-way ANOVA. Scale bar: 50 µm. (**C**) Multinucleated ratio in egg chambers depleted for the regulators of cell–cell and cell–matrix interactions that enhanced multinucleation in the Anillin RNAi modifier screen (Fig. 1D–F), in comparison with control (UAS-mCherry). Depletion of these molecules on their own (in an unperturbed cytokinetic background) does not cause multinucleation. Each dot represents the Multinucleation Ratio of an analyzed egg chamber. Median is indicated. The dashed line represents the mean value of the multinucleation ratio for co-expression of Anillin RNAi with the control line (UAS-mCherry). (**D**) Frequency of multinucleated cells in egg chambers either co-expressing UAS-Dg RNAi with UAS-mCherry RNAi (UAS titulation control), UAS-Tum RNAi with UAS-mCherry RNAi or UAS-Tum RNAi with UAS-Dg RNAi. UAS-driven depletion of Tum causes strong multinucleation frequency in the follicular epithelium, which is significantly increased by the concomitant depletion of Dg. Violin plots and median (magenta) is indicated. Each dot represents an egg chamber. Sample size (n) is indicated on top. *P* value calculated by non-parametric unpaired Mann–Whitney test.

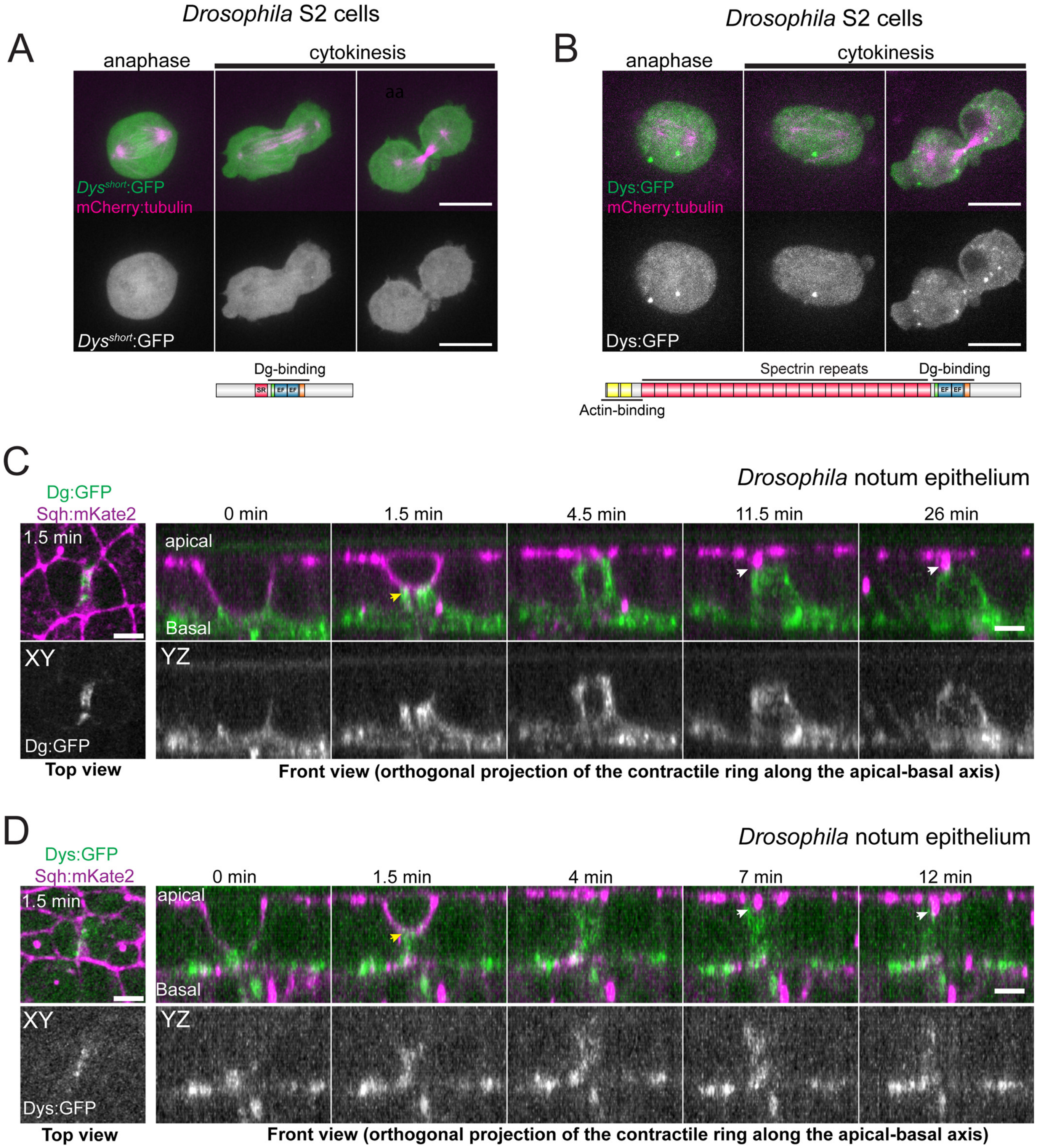

Figure EV4. Dynamic redistribution of the DAPC during epithelial cytokinesis in *Drosophila* S2 cells and pupal notum epithelium.

(**A**, **B**). The short Dys isoform E (**A**) and the long isoform H (**B**) do not show any local accumulation during cytokinesis in *Drosophila* S2 cells. Dys isoforms are GFP-tagged versions (green) and mCherry:Tubulin labels microtubules (magenta). Scale bars: 10 μm. (**C**, **D**). Redistribution of endogenously tagged Dg:GFP (**A**) and Dys:GFP (**B**) during epithelial cytokinesis in the *Drosophila* pupal dorsal thorax (notum). During ring (labeled with Sqh:mKate2) constriction, Dg:GFP (**A**) and Dys:GFP (**B**) accumulate at the basal part of the ingressing membrane (yellow arrows). After ring closure, both proteins become enriched close to the midbody (white arrows). Scale bars: 5 μm.

 © The Author(s)

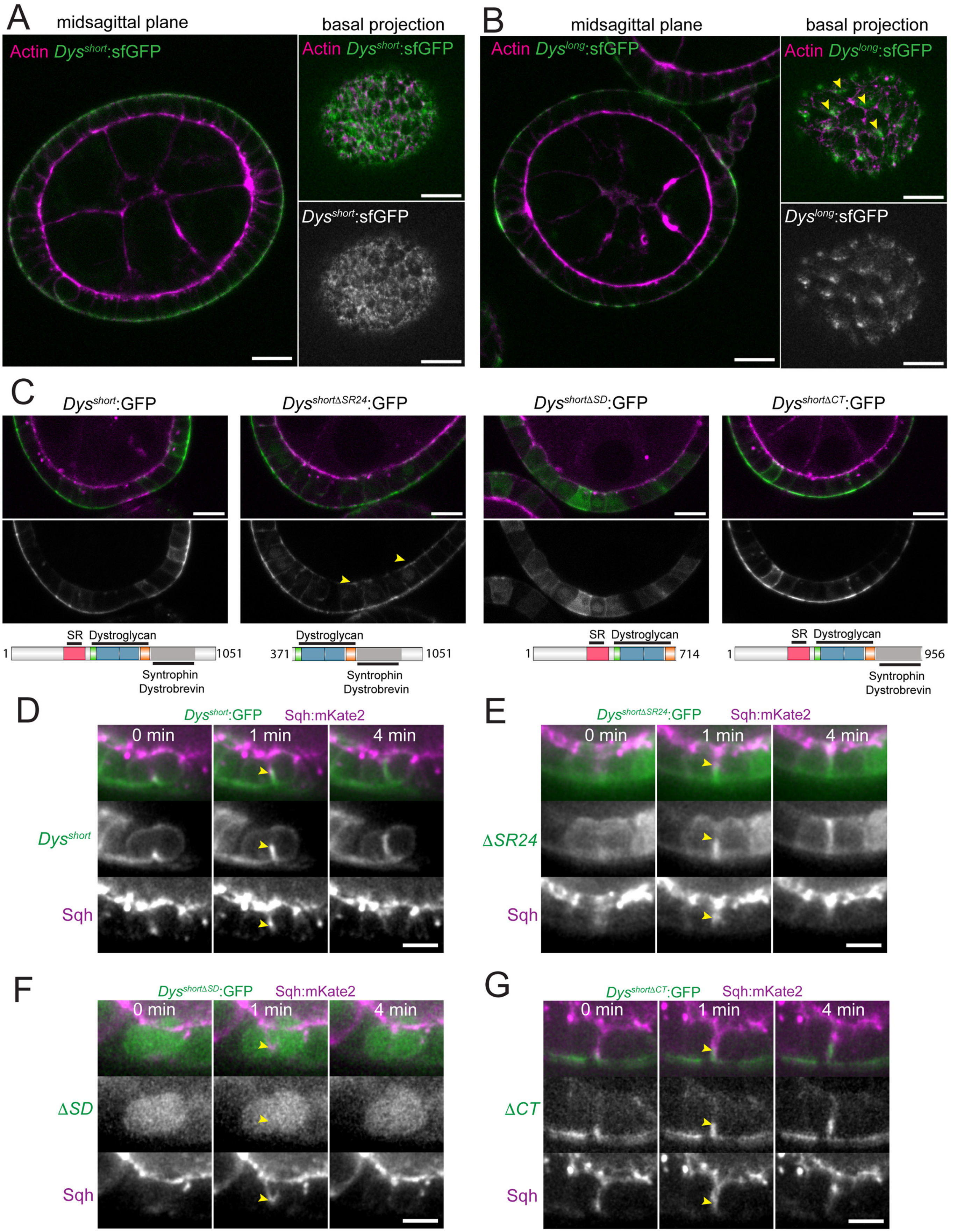
A
midsagittal plane
basal projection
Actin Dysshort:sfGFP
Dysshort:sfGFP
B
midsagittal plane
basal projection
Actin Dyslong:sfGFP
Dyslong:sfGFP
C
Dysshort:GFP
DysshortΔSR24:GFP
DysshortΔSD:GFP
DysshortΔCT:GFP
SR Dystroglycan
1 1051
Syntrophin Dystrobrevin
Dystroglycan
371 1051
Syntrophin Dystrobrevin
SR Dystroglycan
1 714
SR Dystroglycan
1 956
Syntrophin Dystrobrevin
D
Dysshort:GFP Sqh:mKate2
0 min 1 min 4 min
Dysshort
Sqh
E
DysshortΔSR24:GFP Sqh:mKate2
0 min 1 min 4 min
ΔSR24
Sqh
F
DysshortΔSD:GFP Sqh:mKate2
0 min 1 min 4 min
ΔSD
Sqh
G
DysshortΔCT:GFP Sqh:mKate2
0 min 1 min 4 min
ΔCT
Sqh

Figure EV5. Spatial distribution of Dystrophin isoforms and truncated versions.

(**A**) Midsagittal images (left) and basal projection (right) of the proliferative follicular epithelium endogenously expressing Dys^{short}:sfGFP (green) and stained for actin (magenta). Dys^{short} mainly localizes at the basal domain of epithelial cells. Scale bar: 10 µm. (**B**) Midsagittal images (left) and basal projection (right) of the proliferative follicular epithelium endogenously expressing Dys^{long}:sfGFP (green) and stained for actin (magenta). Dys^{long} mainly localizes to the basal domain in a planar polarized manner. Scale bar: 10 µm. (**C**) Midsagittal images of egg chambers expressing UAS-driven Dys^{short}:GFP, $Dys^{short\Delta SR24}$:GFP, $Dys^{short\Delta SD}$:GFP or $Dys^{short\Delta CT}$:GFP and expressing Sqh:mKate2 (magenta). The short *Dys* isoform mainly localizes at the basal domain of epithelial cells, which is not altered by the deletion of the most downstream C-term region ($Dys^{short\Delta CT}$). Partial mislocalization to the apical domain (yellow arrows) is observed in egg chambers expressing $Dys^{short\Delta SR24}$:GFP. Deletion of syntrophins/Dystrobrevin interaction sites leads to Dys mislocalization and only weak basal enrichment ($Dys^{short\Delta SD}$). Note that these UAS-driven constructs show mosaic expression levels in the follicular epithelium. Scale bar: 10 µm. (**D–G**) Time-lapse images of follicle cells expressing UAS-driven Dys^{short}:GFP, $Dys^{short\Delta SR24}$:GFP, $Dys^{short\Delta SD}$:GFP or $Dys^{short\Delta CT}$:GFP. Dys^{short} (**A**) becomes enriched at the ingressing membrane during ring constriction, which is labeled with Sqh:mKate2 (position of the basal part of the ring is marked with arrows). $Dys^{short\Delta SR24}$ (**E**) and $Dys^{short\Delta CT}$ (**G**) present a similar cytokinetic redistribution. In contrast, removal of the syntrophins/Dystrobrevin binding sites prevents Dys accumulation in the ingressing membrane during epithelial cytokinesis (**F**). Scale bar: 5 µm.

© The Author(s)
